# Protocol for constructing and characterizing recombinant vectored vaccines for rabies virus

**DOI:** 10.1016/j.xpro.2024.103392

**Published:** 2024-10-17

**Authors:** Manar E. Khalifa, Muhammad Munir

**Affiliations:** 1 Division of Biomedical and Life Sciences, Faculty of Health and Medicine, Lancaster University, Lancaster LA1 4YG, UK

**Keywords:** Flow Cytometry, Health Sciences, Immunology, Microbiology, Molecular Biology, Antibody, Biotechnology and bioengineering

## Abstract

Recombinant vectored vaccines provide economical, rapidly engineered, and tailored immunization strategies. Here, we present a protocol for constructing and characterizing recombinant vectored vaccines for rabies virus (RV). We describe steps for generating replication-competent vesicular stomatitis virus (rcVSV) by replacing vesicular stomatitis virus (VSV) glycoprotein (G) with RV G gene and for recovering rcVSV by co-transfecting baby hamster kidney (BHK-21) cells with the antigenomic VSV cDNA plasmid (pVSV). We then detail procedures for the functional, structural, and molecular characterization of generated rcVSV-RV-Gs.

## Highlights

• Steps to display heterologous viral glycoproteins on recombinant VSVs
• Procedures for rescuing recombinant VSV in mammalian cells
• Instructions to evaluate the replication fitness of rcVSV-RV-G
• Steps for assessing the expression of RV-G and VSV-M proteins by western blot

## Abstract (editor highlights)

Publisher’s note: Undertaking any experimental protocol requires adherence to local institutional guidelines for laboratory safety and ethics.

## Graphical abstract

**Figure:**
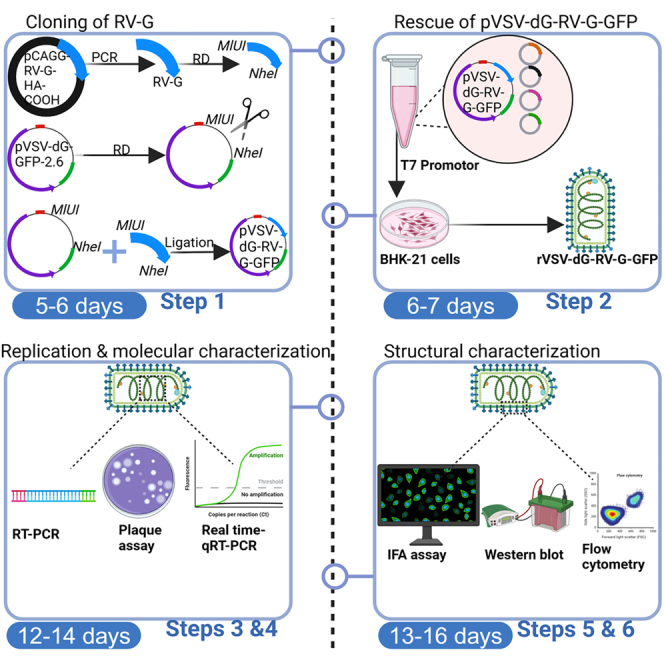

## Before you begin

Vesicular stomatitis virus (VSV), the prototype member of the family *Rhabdoviridae,* is a non-segmented, negative strand RNA virus. The VSV genome is 11-kb, and it encodes five structural genes including: nucleoprotein (N), phosphoprotein (P), matrix (M), glycoprotein (G), and large polymerase (L).^1^ Owing to its capability of stable maintenance and expression of transgenes, VSV has been demonstrated as an efficient system for generating recombinant vaccines against viruses of animal and human origins.^2^ Additionally, VSV offers a versatile tool to study virus host interactions due to its wide host spectrum.^3^ Rabies is a highly fatal disease, caused by rabies virus (RV), and is responsible for approximately 59,000 human deaths annually.^4^ The remarkably diverse reservoir species of RV render its control and prevention challenging. However, a substantial decrease in human rabies cases could be achieved through development of novel vaccines targeting RV reservoirs. To study the role of the RV-G in RV entry and its interaction with the cellular receptors. Herein, we describe a reverse genetics system based on the generation of VSV in which the G gene of VSV is replaced by a reporter gene (green fluorescent protein, GFP) together with the heterologous RV-G gene.

**Figure 1:**
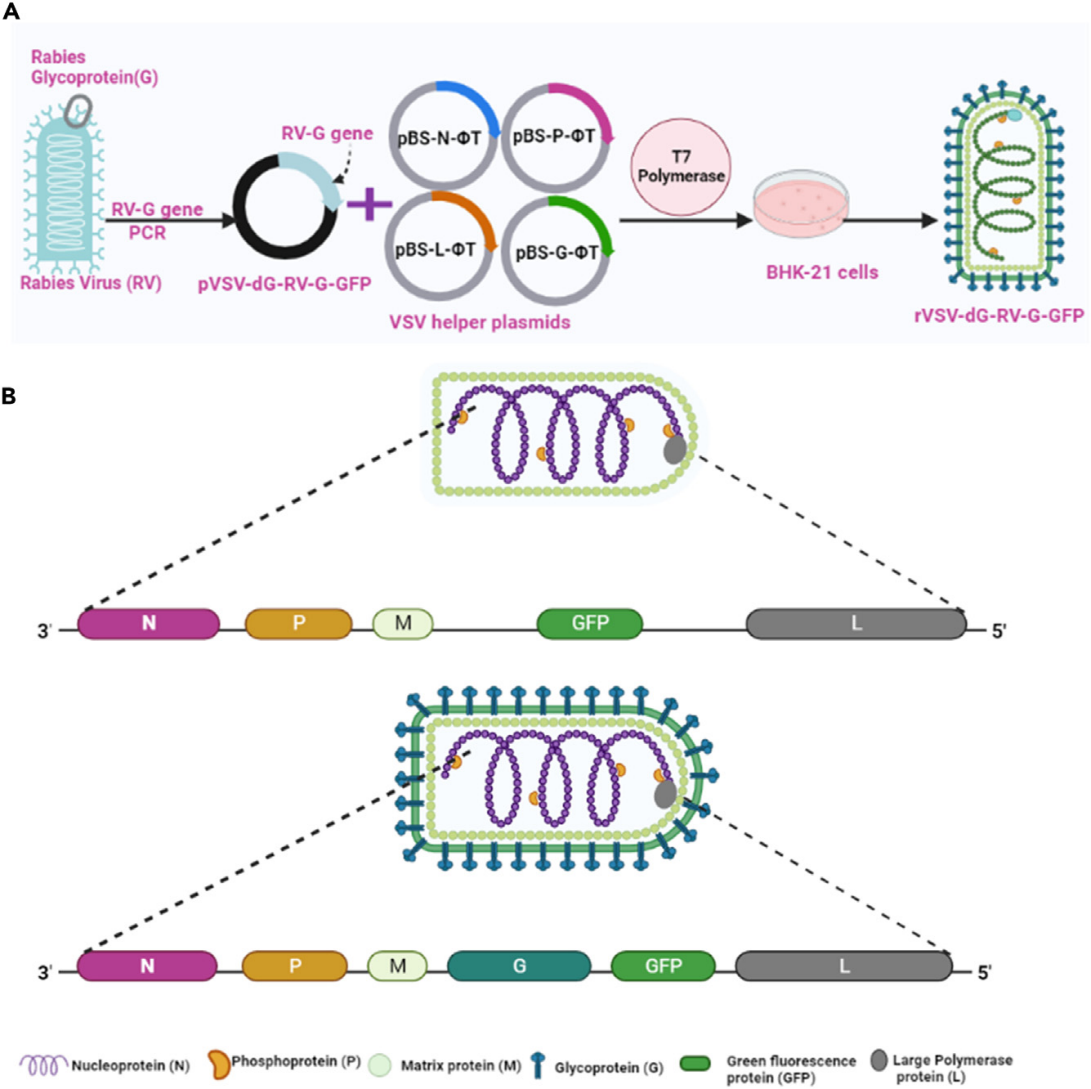
Schematic diagrams demonstrating the rVSV plasmids and the generation process of rVSV-dG-RV-G-GFP from pVSV-dG-RV-G-GFP (A) Schematic representation of the generation process of rVSV-dG-RV-G-GFP, including RV-G gene cloning from pCAGG RV-G-GHA plasmid into the pVSV-dG-GFP-2.6 plasmid, followed by infection of BHK-21 cells with rFPV-T7, and co transfection with pVSV-dG-RV-G-GFP, and VSV-system helper plasmids, resulting in rVSV-dG-RV-G-GFP production. (B) Schematic diagrams showing the genomic organization of the pVSV-dG-GFP-2.6 (top diagram) and rVSV-dG-RV-G-GFP (bottom diagram). Abbreviations: N; nucleoprotein, P; phosphoprotein, M; matrix, G; glycoprotein, GFP, green fluorescent protein, L, large polymerase.

**Figure 2:**
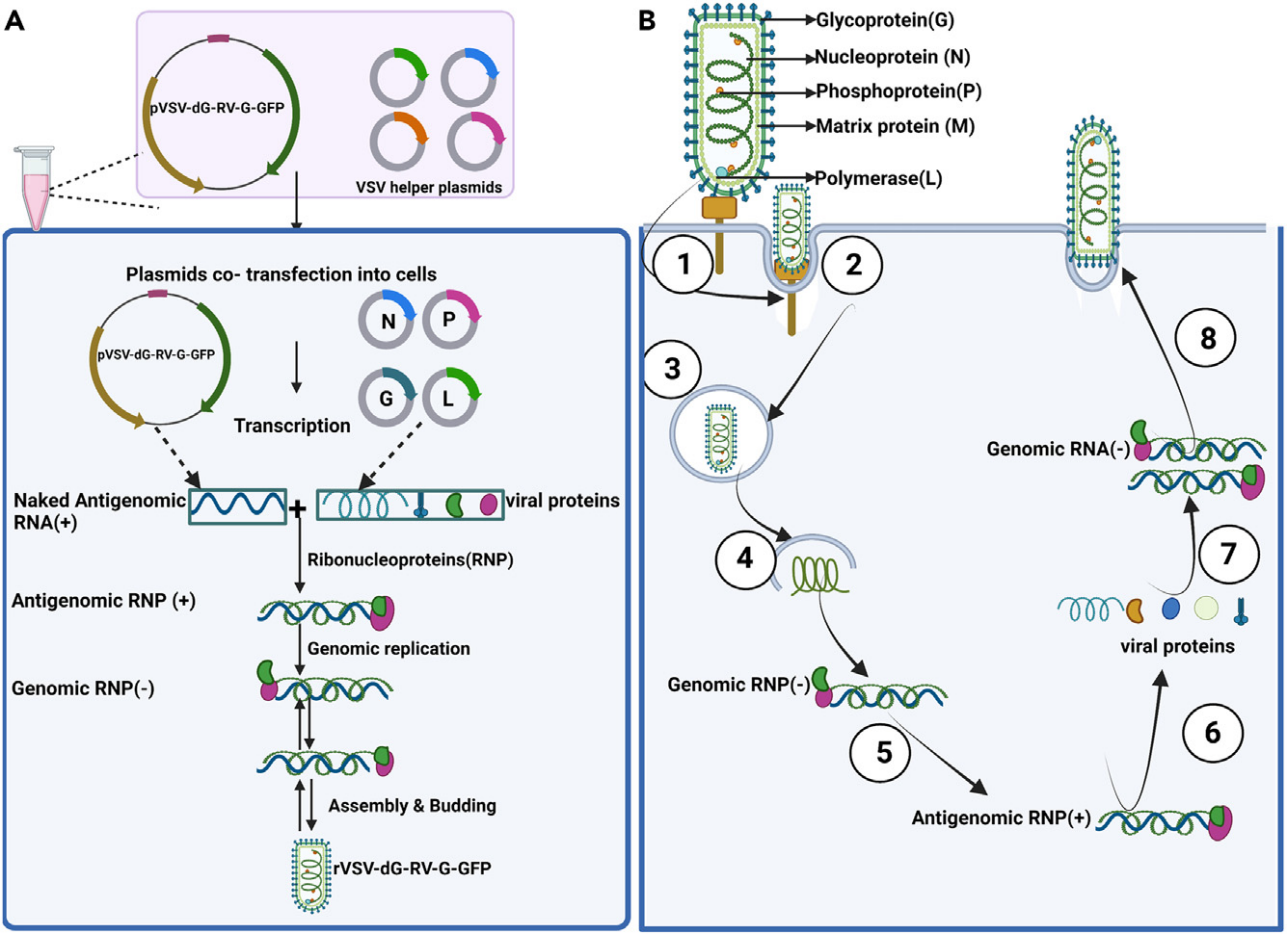
Replication of rVSV-dG-RV-G-GFP and rabies virus (A) Overview of the rescue process for rVSV-dG-RV-G-GFP, including co-transfection of pVSV-dG-RV-G-GFP and VSV helper plasmids to produce the modified VSV antigenome, which is then encapsulated by viral proteins to form ribonucleoprotein (RNP) complexes, followed by replication, assembly, and budding of recombinant virions. (B) Natural replication cycle of rabies virus: (1) virus binding to cellular receptors,(2) internalization via receptor-mediated endocytosis, (3) fusion of viral and endosomal membranes at low pH conditions, (4) release of viral RNA, (5) transcription of genomic RNP into antigenomic RNP, (6) translation of viral proteins, (7) replication of RNA genomes using antigenomic RNP as a template, (8) assembly of viral proteins and RNA genomes and release of new virions by budding.

This protocol describes the sequential process to successfully manipulate the VSV genome and to clone transgenes in compatibility (Figure 1A). The pVSV-dG-GFP-2.6 vector which encodes the antigenomic cDNA of VSV, can be manipulated to insert a transgene (exemplified here with full-length open reading frame (ORF) of the rabies virus surface glycoprotein (RV-G), derived from the Egyptian strain isolate (GenBank accession number: MK760770.1) (Figure 1B). The RV-G is inserted in the multiple cloning sites of pVSV-dG-GFP-2.6 (recipient plasmid) between the M and the GFP genes using unique MluI and NheI restriction sites. VSV recovery then involves the co-transfection of the full length antigenomic viral RNA of VSV, encoding the RV-G (pVSV-dG-RV-G-GFP) along with plasmids encoding the viral ribonucleoprotein complex (RNP). The RNP is formed by P and L along with N proteins. Upon co-transfecting the RNP complex with the antigenome viral RNA, the RNA is transcribed by T7 polymerase; supplied by infecting the cells with the recombinant fowl pox virus (rFPV). Consequently, the translation of encoded proteins occurs, allowing the assembly of nucleoprotein around the antigenomic RNA and polymerase to replicate forming RNP containing the genomic RNA. The assembly of the infectious virus occurs following the transcription of the mRNA from the genomic RNP and its translation^5^^,^^6^ (Figure 2). The M protein required for assembly and budding is not provided in trans, since it is produced from the virally encoded M gene released from the generated infectious viral particles.^7^^,^^8^ Since all the DNA constructs are under the control of T7 RNA polymerase promoter, the rFPV is utilized to infect cells as a source of T7 RNA polymerase in trans. The efficiency of T7 polymerase promoter infection is assessed by transfecting the BHK-21 cells with pCITE GFP plasmid, which encodes GFP under the control of T7 promoter.^7^ Collectively, this protocol provides a detailed method for the generation of a rcVSV carrying a transgene of RV. The rcVSV has the potential to serve as a recombinant vaccine and facilitates the study of virus host interactions.

### Biosafety

Since VSV is a biosafety level 2 pathogen, all procedures which involves handling of VSV should be carried out in a certified biological safety cabinet in designated labs. Thus, before starting, it is necessary to obtain all required documentation to accomplish biosafety level 2 (BSL-2) work.

**Figure 3:**
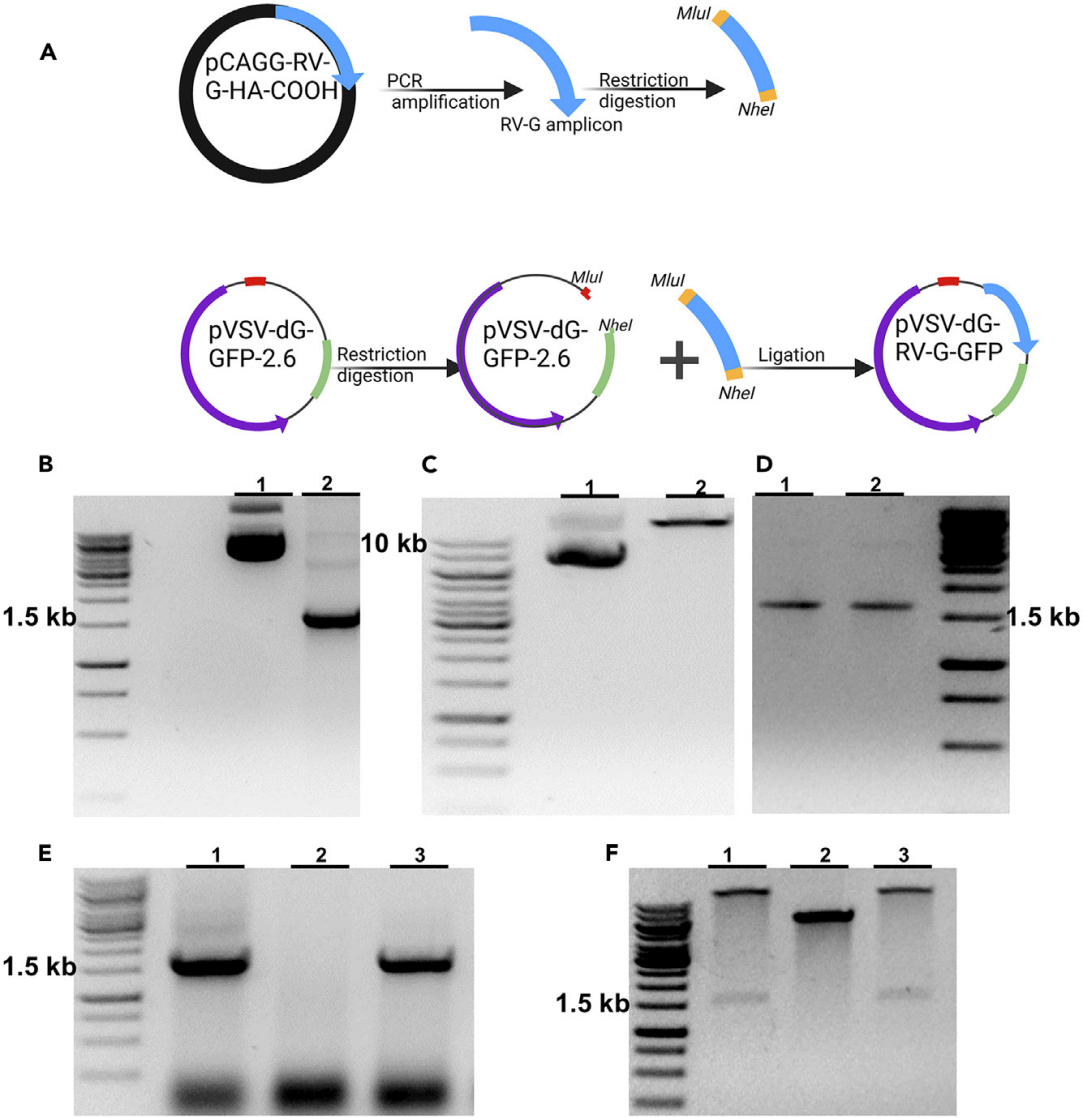
Cloning of RV-G into pVSV-dG-GFP-2.6 backbone (A) Schematic diagram illustrating the cloning process of RV-G into the pVSV-dG-GFP-2.6. (B) Agarose gel electrophoresis showing the amplification of RV-G from pCAGG RV-G HA plasmid using primers with MluI and NheI restriction sites, (1) pCAGG-RV-G-HA-vector and (2) RV-G PCR product (∼ 1.5 kb). (C) Agarose gel electrophoresis showing restriction digestion of pVSV-dG-GFP-2.6 with MluI-HF and NheI-HF enzymes; (1) uncut pVSV-dG-GFP-2.6 ( ∼ 13.4 kb) and (2) linearized vector( ∼ 13.4 kb). (D) Agarose gel electrophoresis showing restriction digestion of RV-G PCR amplicon with MluI-HF and NheI-HF restriction enzymes, (1,2) linearized RV-G amplicons (1.5 kb). (E) Agarose gel electrophoresis of colony PCR products showing positive colonies (1) and (3), while (2) showed no insert. (F) Agarose gel electrophoresis showing the diagnostic restriction digestion of purified pVSV-dG-RV-G-GFP plasmid using MluI and NheI enzymes, the pVSV-dG-GFP-2.6 backbone (13.4 kb) and the RV-G insert (1.5 kb) shown in the digested plasmid of plasmids (1) and (3), while the plasmid (2) showed no insert.

**Table 1:** Primers used for RV-G gene cloning in the pVSV-dG-GFP-2.6 backbone

RV-G cloning primers	Source
RV-G F: TGTTT*ACGCGT*CACT**ATGGTGCCCCAGGCCCTGCT**	Invitrogen, Thermo Fisher Scientific
RV-G R: ATGAAGAATCTG*GCTAGC***AGGATTTGAGTTACAGCCGTGTCTCGCCCCCGCTCTT**	Invitrogen, Thermo Fisher Scientific

### Plasmids

**Timing: 4–5 days**

1. To generate the pCAGG-RV-G-HA-COOH vector (plasmid encoding the full-length RV-G, serves as the donor plasmid for cloning the RV-G into pVSV-dG-GFP-2.6):
  a. Retrieve the full codon sequence of RV-G gene from NCBI (GenBank: NM_760770.1, MK760770).
  b. Codon optimize (performed by the Bio Basic, UK in this study) the RV-G sequence for *Homo sapiens* and clone the synthesized RV-G in pCAGG vector with HA tag at the C-terminus, referred to as pCAGG-RV-G-HA-COOH plasmid.
2. For rcVSVs rescue, the following plasmids are required:
  a. pVSV-dG-RV-G-GFP-2.6.
  b. pBS-P-ФT.
  c. pBS-L-ФT.
  d. pBS-N-ФT.
  e. pBS-G-ФT.
3. Transform each of these plasmids into *DH5α E. coli* competent cells using the heat shock transformation procedure.
  a. Thaw 50 μL of *DH5α E. coli* competent cells from −80°C on ice.
  b. Add 50 ng of plasmid DNA into the competent cells and mix gently by flicking the tube. Incubate for 15 minutes (min) on ice.
  c. For heat shock, transfer the transformation mixture to 42°C for 45 seconds (s) using a heat block. Immediately, place the cells back on ice for 10 min.
  d. Add 250 μL of prewarmed SOC medium to the transformation mixture. Incubate the cells at 37°C for 1 hour (hr) in the shaking incubator.
  e. Plate 60 μL of culture into LB agar plates with sterile cell spreader using the streaking plate method.
4. Grow the transformed bacteria on LB agar plates, supplemented with ampicillin (100 μg/mL) for 16 h at 37°C.

**CRITICAL:** If no colonies are found, prepare duplicate LB agar plates of each plasmid, incubate one of them at 30°C for 24–48 h (since cloned DNA might be toxic for the cells, so incubation at lower temperature can mitigate toxicity through lowering plasmid copy number) and incubate the other plate at 37°C for 18 ± 2 h.

5. Pick a single colony and incubate it in 100 mL LB media, supplemented with 100 μg/mL ampicillin for 18 ± 2 h, at 37°C in a shaker incubator.
6. Lyse the bacteria, extract, and purify the plasmid DNAs using the Qiagen midi prep kit following the manufacturer’s protocol instructions.
7. Determine the concentration of the plasmid DNAs using the nanodrop.

### Primer and probe design

**Timing: 4 days**

8. For cloning the full codon optimized sequence of RV-G in the multiple cloning site of pVSV-dG-GFP-2.6 (flanking the region between M and GFP genes using two restriction sites *MluI* and *NheI)* (Figure 3A).
  a. Design the forward and reverse primers as shown in Table 1:
    i. The forward primer (RV-G F) contains *MluI* site (underlined and italic letters) and the first 20 nucleotides of the coding region of RV-G gene (bold letters). The 4 nucleotides before the restriction sites represent the complementary bases to the vector sequence.
    ii. The reverse primer (RV-G R) contains the last 27 nucleotides of the G gene (bold letters), followed by *NheI* site (underlined italic letters).
9. To confirm the stability of RV-G insert after RV-G cloning into pVSV-dG-GFP-2.6 and upon rVSV-dG-RV-G-GFP rescue, perform RT-PCR.
  a. Design primers flanking the RV-G insert in the rVSV-dG-RV-G-GFP.
    i. Design the forward primer (VSV-up) so as to encompass the last 33 nucleotides of M gene.
    ii. Design the reverse primer (VSV-down) so as to flank the first 39 nucleotides of GFP gene.
10. To assess the growth kinetics of the generated rVSV-dG-RV-G-GFP relative to the rVSV-GFP-WT (wild type VSV virus with GFP, serves as control, obtained from the host lab), employ the absolute qPCR method. This allows the comparative quantification of viral genomic RNA copies between rVSV-dG-RV-G-GFP and rVSV-GFP-WT.
  ***Note:*** The VSV N gene is targeted since VSV genome RNA replication is proportional to the amount of N protein synthesized.^9^
  a. Design and synthesize a VSV N probe, considering the following parameters.^10^
    i. Melting temperature (Tm) of the probe to be 8°C–10°C above Tm of the designed primers.
    ii. Avoid primer dimer, design the probe length to be between 18-30 bases.
    iii. Ensure that the amplification product length does not exceed 150 bp to maintain high qPCR efficiency.
    iv. Use BLAST to verify the specificity of the amplified region.
    v. The following website can be used to validate the designed primer/probe.
    vi. Synthesize and label the designed probes with FAM and TAMRA at the 5′ end and 3′ ends, respectively.

### Cell line maintenance

**Timing: 2–3 days**

11. Revive the frozen BHK-21 cell line.
  a. Transfer the BHK-21 cells cryovial from liquid nitrogen and thaw it immediately at 37°C.
  b. Upon thawing, slowly add 10 mL of pre-warmed growth medium (DMEM, high glucose with GlutaMAX, supplemented with 10% FBS, and 1× antibiotic antimycotic solution) to the cell suspension and centrifuge at 200 × *g* for 5 min.
  c. Discard the supernatant and resuspend the pellet in pre-warmed growth medium.
  d. Incubate the cells in cell culture flasks at 37°C with 5% CO_2_.
  e. Passage the BHK-21 cells 2–3 times /week, when they reach 80%–90% confluency.
12. Maintain BHK-21 cells in DMEM with GlutaMAX, supplemented with 10% FBS, and 1× antibiotic antimycotic solution.
13. For the BHK-21 cell line cryopreservation.
  a. Detach cells from the culture flask with 1× trypsin.
  b. Centrifuge the cells for 5 min at 200 × *g*, then resuspend the cell pellet in 1 mL of ice-cold freezing medium (10% DMSO in FBS) in cryovials.
  c. Store the cryovial in liquid nitrogen until use.

## Key resources table

**Table:** 

REAGENT or RESOURCE	SOURCE	IDENTIFIER
**Antibodies**		

Mouse RV glycoprotein antibody (western blot dilution, 1:2,000), (IFA dilution 1:400)	Bio-Rad	Cat# MCA2828
Mouse VSV M antibody (western blot dilution, 1:2,000), (IFA dilution 1:400)	Abcam	Cat# EB0011
Goat anti-mouse IgG (H + L) secondary antibody, HRP (dilution 1:3,000)	Abcam	Cat# ab6789
Alexa Fluor goat anti-mouse IgG (488) (dilution 1:800)	Invitrogen	Cat# A11001

**Bacterial and virus strains**		

MAX Efficiency DH5α competent cells	Thermo Scientific	Cat# 18258012
rVSV-dG-RV-G-GFP	This study	N/A

**Chemicals, peptides, and recombinant proteins**		

Acrylamide/Bis solution (30%)	Bio-Rad	Cat# 1610158
Agarose low EEO	NBS Biologicals, Cambridge	Cat# R1040
Ammonium persulfate (APS)	Bio-Rad	Cat# 1610700
Ampicillin-Na-salt	Sigma-Aldrich	Cat# A9518
Antibiotic-Antimycotic (100×)	Gibco, Life Technologies	Cat# 15240062
BSA (albumin bovine fraction V)	Sigma-Aldrich	Cat# 05482
Carboxy-methyl-cellulose sodium salt	Sigma-Aldrich	Cat# C4888-500G
Crystal violet	Sigma-Aldrich	Cat# C0775
DAPI	Thermo Scientific	Cat# 62247
DMEM, high glucose, GlutaMAX Supplement pyruvate	Gibco, Thermo Fisher Scientific	Cat# 10569010
Dimethyl sulfoxide (DMSO)	Fisher Scientific	Cat# 175462
EDTA	Millipore	Cat# 324503
Ethanol	Fisher Scientific	Cat# 2107463
Fetal bovine serum	Gibco, Life Technologies	Cat# 10500-64
GelRed nucleic acid stain 10,000× water	Millipore	Cat# SCT123
GeneRuler 1 kb DNA ladder	Thermo Scientific	Cat# SM0311
HEPES buffer (1 M)	Gibco	Cat# 15630080
LB Agar (Lennox L Agar), powder	Invitrogen, Thermo Fisher Scientific	Cat# 22700025
L-glutamine (200 mM)	Gibco, Life Technologies	Cat# 25030-081
LIVE/DEAD Fixable Violet Dead Cell Stain Kit	Thermo Fisher Scientific	Cat# L34964
MEM (10 X)	Gibco, Life Technologies	Cat# 21430-020
Methanol	Fisher Scientific	Cat# 2196137
MluI-HF	New England Biolabs	Cat# R3198S
NheI-HF	New England Biolabs	Cat# R3131S
Non-essential amino acid solution (100×)	Gibco, Life Technologies	Cat# 11140050
NP-40	Thermo Scientific	Cat# 85124
Nuclease-free water	Thermo Scientific	Cat# 10977-035
NuPAGE (transfer buffer)	Novex, Life Technologies	Cat# 2270643
Opti-MEM	Gibco, Life Technologies	Cat# 31985-070
Paraformaldehyde (4%)	Thermo Scientific	Cat# J19943-k2
Permeabilization buffer (10 X)	Thermo Scientific	Cat# 00833356
Pierce Protease inhibitor tablet	Thermo Scientific	Cat# A32963
Pierce ECL western blotting substrate	Thermo Scientific	Cat# 32106
Potassium phosphate dibasic	Sigma-Aldrich	Cat# P0662
Pre-stained protein ladder (10–180 kDa)	Abcam	Cat# ab116027
Q5-high fidelity DNA polymerase	New England Biolabs	Cat# M0491S
SDS sample buffer	Life Technologies	Cat# 1597380
SDS solution 10%	Bio-Rad	Cat# 1610416
Skimmed milk powder	Millipore	Cat# 70166
SOC medium	New England Biolabs	Cat# B9020S
Sodium bicarbonate solution (7.5%)	Sigma-Aldrich	Cat# S5761
Sodium chloride	Sigma-Aldrich	Cat# S5886
Sodium dodecyl sulphate (SDS)	Sigma-Aldrich	Cat# L3771
Sodium hydroxide	Sigma-Aldrich	Cat# 221465
Sodium phosphate dibasic	Sigma-Aldrich	Cat# S5136
T4 DNA ligase	New England Biolabs	Cat# M0202
TEMED	Bio-Rad	Cat# 1610801
Tris-base	Sigma-Aldrich	Cat# 252859
Tris-EDTA 1×	Fisher Scientific	Cat# BP2473
Triton X-100	Sigma-Aldrich	Cat# T8787
Trizma hydrochloride	Sigma-Aldrich	Cat# RDD009
Trypsin 2.5%	Gibco, Thermo Fisher	Cat# 15090-046
TurboFect Transfection Reagent	Thermo Scientific	Cat# R0532
Tween 20	Sigma-Aldrich	Cat# P2287
VECTASHIELD antifade mounting buffer	Vector Laboratories	Cat# ZH1108
β-Mercaptoethanol	Bio-Rad	Cat# 1610710

**Critical commercial assays**		

QIAamp Viral RNA mini kit	QIAGEN	Cat# 52906
GeneJET Plasmid Midiprep Kit	Invitrogen, Thermo Fisher Scientific	Cat# K0482
QIAprep Spin Miniprep kit	QIAGEN, Germany	Cat# 27106
Gene JET Gel Extraction Kit	Thermo Fisher	Cat# 01237174
SuperScript IV Reverse Transcriptase	Invitrogen, Thermo Fisher Scientific	Cat# 18090010
SuperScript III Platinum One-Step RT-qPCR	Invitrogen, Thermo Fisher Scientific	Cat# 11732088

**Experimental models: Cell lines**		

BHK-21 cells	ATCC	ATCC No: CCL-10

**Oligonucleotides**		

VSV-UP: GCCCACCATGGGAGCGTGG GTCCTGGATTCTATCAGCCACTTC	Invitrogen, Thermo Fisher Scientific	N/A
VSV down: TGGGACAACTCCAGTGAA AAGTTCTTCTCCTTTACTCAT	Invitrogen, Thermo Fisher Scientific	N/A
qPCR-VSV N-F: TGATCGACTTTGGATTGTCTTCTAA	Invitrogen, Thermo Fisher Scientific	N/A
qPCR-VSV-N R: TCTGGTGGATCTGAGCAGAAGAG	Invitrogen, Thermo Fisher Scientific	N/A
qPCR-VSV-N probe: FAM-ATATTCTTCCG TCAAAAACCCTGCCTTCCA-TAM	Invitrogen, Thermo Fisher Scientific	N/A
Random hexamers (100 mL)	Invitrogen, Thermo Fisher Scientific	Cat# N8080127

**Recombinant DNA**		

pBS-P-ФT	Kerafast	Cat# EH1014
pBS-L-ФT	Kerafast	Cat# EH1015
pBS-N-ФT	Kerafast	Cat# EH1013
pBS-G-ФT	Kerafast	Cat# EH1016
VSV-dG-GFP-2.6	Kerafast	Cat# EH1026
pCAGG-GFP	A gift from Luis Martinez-Sobrido, Texas Biomedical Research Institute, USA	N/A
pCITE-GFP	A gift from Luis Martinez-Sobrido, Texas Biomedical Research Institute, USA	N/A
pCAGG-RV-G-HA-COOH	This study	N/A
pVSV-dG-RV-G-GFP	This study	N/A

**Software and algorithms**		

ZEN Microscopy software	Carl Zeiss Imaging	(blue) 3.6
GraphPad prism	GraphPad Software, Inc.	Version 9
CFX Maestro Software	Bio-Rad	Version 3.1

**Other**		

0.2 mL PCR tubes	Applied Biosystems	N/A
0.45-, 0.2 μm filter	Starlab	Cat# E4780-1456
1.5 mL microcentrifuge tubes	Merck	Cat# HS4323
6-Well cell culture plate	Greiner	Cat# 657160
Autoclave	Astell	N/A
Bacterial incubator 37°C	Sanyo	N/A
Blotting papers	Bio-Rad	Cat# 170396
Cell culture CO_2_ incubator	Panasonic	N/A
Cell culture flask with filter cap 75 cm^2^	Greiner	Cat# 658175
Centrifuge 5424 R	Eppendorf	N/A
Centrifuge Allegra X-30R	Beckman Coulter	N/A
Centrifuge tube 50 mL	Greiner	Cat# 227261
CFX96 Real-Time system	Bio-Rad	N/A
ChemiDoc MP imaging system	Bio-Rad	N/A
Cryovials, 1.8 mL	Corning	N/A
CytoFLEX flow cytometer	Beckman Coulter	N/A
Latex gloves	Fisher Scientific	N/A
Nano-Drop 2000c spectrophotometer	Thermo Scientific	Cat#ND2000CLAPTOP
Nunc Thermanox coverslips	Thermo Scientific	Cat# 174942
Parafilm	Starlab	Cat# 13080
Petri dishes for bacteria, 100 mm	Sarstedt	N/A
Pipette tips 1 mL, 200 μL, 10 μL	Starlab	N/A
Pipettes 200 μL–1 mL, 20–200 μL, and 0.5 mL–1 mL	Gilson	N/A
PTC-200 Peltier thermal cycler	Universal Resource Trading Ltd.	N/A
PVDF membrane	Thermo Scientific	Cat# 88518
qPCR tube 0.1 mL	Bio-Rad	N/A
SDS-PAGE system	Bio-Rad	N/A
Stripette, 5, 10, 25 mL	Corning	N/A
Trans-blot turbo membrane blotter	Bio-Rad	N/A
UV transilluminator	Syngene	N/A
Vortex	SLS Lab Basics	N/A
ZOE fluorescent cell imager	Bio-Rad	N/A

## Materials and equipment

**Table:** Plaque overlay medium

Reagent	Amount (500 mL)
Sterile bottled water	284 mL
10× MEM	100 mL
Sodium Bicarbonate Solution (7.5%)	30 mL
L-glutamine/Gluta-Max	10 mL
Non-Essential Amino Acids	10 mL
HEPES (1 M)	26 mL
Fetal bovine serum	40 mL

***Note****:* Firstly prepare 500 mL of 3% Carboxy methyl cellulose (CMC) in Milli-Q water, then autoclave. Keep the autoclaved medium at 22°C–25°C to cool down, then place the CMC solution at 4°C for 18 ± 2 h to dissolve. On the day of plaque assay, add the above components (500 mL) to the dissolved 3% CMC (500 mL) solution and allow to mix using magnetic stirrer.

Store at 4°C.

**Table:** 1× Phosphate Buffered Saline (PBS) (pH, 7.4)

Reagent	Final concentration	Amount (1 L)
NaCl (molecular weight; mw: 58.44 g/mol)	0.137 M	8 g
KCl (mw: 74.551 g/mol)	0.0027 M	0.2 g
Na_2_HPO_4_ (mw: 141.96 g/mol)	0.01 M	1.44 g
KH_2_PO_4_ (mw: 136.086 g/mol)	0.0018 M	0.245 g
Milli-Q water	–	Up to 1 L

Heat the mixture until all the components dissolve, then titrate until the pH reaches 7.4.

Store at 20°C–25°C for up to 1 year.

**Table:** 10× Sodium dodecyl sulfate (SDS) Running Buffer

Reagent	Final concentration	Amount (1 L)
Tris base (mw: 121.14 g/mol)	0.2501 M	30.3 g
Glycine (mw: 75.07 g/mol)	1.924 M	144.4 g
SDS (mw: 288.38 g/mol)	0.03467 M	10 g
Milli-Q water	Up to 1 L	–

**CRITICAL:** SDS is flammable and may cause respiratory tract irritation. Thus, it should be handled in a chemical hood with gloves, eyeglasses, and proper PPE.

Place the mixture on a hot plate magnetic stirrer to dissolve the reagents.

Store at 20°–25°C for up to 6 months.

### Agarose gel reagent

**Table:** 10× Tris acetate EDTA (TAE) buffer

Reagent	Amount (1 L)
Tris base	48.5 g
Glacial acetic acid	11.4 mL
0.5 M EDTA	20 mL
Milli-Q water	Up to 1 L

Store at 20°–25°C for up to 1 year.

### Western blot reagents

**Table:** 1% NP-40 lysis buffer (pH 7.4, 100 mL)

Reagent	Final concentration	Amount (100 mL)
10% NP-40	1%	10 mL
0.5 M EDTA	1 mM	1 mL
1 M NaCl	150 mM	3 mL
0.5 M Tris HCl (pH 7.4)	50 mM	2 mL
Milli-Q water	–	Up to 100 mL

Store at 4°C.

• PBS-T, 0.5% tween-20 in 1× PBS (500 mL): In 500 mL bottle, add 450 mL of 1× PBS, then mix with 2.5 mL Tween-20. Add up to 500 mL 1× PBS. Store at 20°–25°C.
• Blocking buffer (5%, 10 mL): Weigh 0.50 g of non-fat dry milk (NFDM), then mix it with 10 mL of 0.5% tween-20 in 1× PBS. Preferably prepare fresh.
• 10% β-mercaptoethanol loading dye (1 mL): Mix 450 μL of 4× loading dye with 450 μL Milli-Q water and add 90 μL β-mercaptoethanol in a microcentrifuge tube.
• 1× Transfer buffer (500 mL): Mix 25 mL of NuPAGE transfer buffer (20×) into 475 mL of Milli-Q water.
• 1.5 M Tris HCl pH 8.8: Weigh 27.23 g of Tris base and add to a flask with magnetic stirrer, complete until 80 mL of Milli-Q water in magnetic stirrer, measure the pH. Then, add HCL about 2 mL each time and measure pH until reaches 8.8. Complete with Milli-Q water until 150 mL.
• 0.5 M Tris HCl pH 6.8: Weigh 18.28 g of Tris base, add in a flask with magnetic stirrer complete until 80 mL Milli-Q water, measure the pH, then, add HCL about 2 mL each time until pH reaches 6.8, then complete up to 150 mL Milli-Q water.
• APS (10%, 10 mL): In 50 mL falcon tube, add 1 g of ammonium persulfate into 10 mL Milli-Q water. Store in 1.5 mL microcentrifuge tubes and keep at −20°C.
• Antibody diluent (5%, 5 mL): In a 50 mL falcon tube, add 0.25 g NFDM in 5 mL 0.5% tween in 1× PBS.

### IFA reagents

• 0.1% Triton X-100 (20 mL): In a 50 mL falcon tube, add 20 μL triton x-100 in 20 mL Milli Q-water, vortex the solution to allow proper mixing.
• Blocking buffer, 0.5% bovine serum albumin (BSA), (50 mL): In a 50 mL conical tube, weigh 0.25 g BSA and add into 50 mL of warm Milli-Q water. Keep the solution on magnetic stirrer to solubilize. Prepare fresh.

### Flow cytometry reagents

• FCS buffer (2% FBS in 1× PBS): In a 50 mL falcon tube, add 1 mL of FBS into 50 mL of Milli Q-water. Prepare fresh.
• 1× Permeabilization Buffer (2 mL): Add 200 μL of 10× permeabilization buffer in 1800 μL of FCS buffer. Prepare fresh.

## Step-by-step method details

### Cloning of RV-G into pVSV-dG-GFP-2.6 expression vector

**Timing: 5–6 days**

The Steps for RV-G cloning into pVSV-dG-GFP-2.6 are outlined in Figure 3A.

1. Day 1: To amplify the RV-G from the pCAGG-RV-G-HA-COOH vector (RV-G donor plasmid), prepare the PCR reaction in 0.2 mL PCR tubes (on ice) using high fidelity (HF) Q5 DNA polymerase enzyme as follows:

**Table:** 

Reagent	Amount (50 μL)
DNA template (pCAGG-RV-G-HA-COOH-vector)	500 ng
Q5 HF DNA Polymerase 0.02 U/ μL	0.5 μL
RV-G-F (10 μM)	2.5 μL
RV-G R (10 μM)	2.5 μL
5× Q5 Reaction Buffer (1×)	10 μL
10 mM dNTPs	10 μL
5× Q5 High GC Enhancer (1×)	10 μL
Nuclease-Free Water (NFW) up to	50 μL

2. Upon setting up the PCR reaction, gently mix the samples in a vortex, then transfer the samples to the thermocycler. Set the thermocycler conditions as follows:

**Table:** 

Steps	Temperature	Time	Cycles
Initial Denaturation	98°C	3 min	1
Denaturation	98°C	30 s	35–40 cycles
Annealing	65°C	1 min	
Extension	72°C	2 min	
Final extension	72°C	10 min	1
Final Hold	4°C	–

**CRITICAL:** It is recommended to set the initial denaturation for 3 min to ensure separation of the double stranded DNA template, allowing primers to bind to the target region and initiate the extension step.

**CRITICAL:** It is recommended to use the NEB Tm calculator to optimize the annealing temperature based on the polymerase enzyme and primers used in the PCR reaction.

3. Upon amplification, perform agarose gel electrophoresis to confirm and purify the amplified PCR product.^11^
  a. To prepare 50 mL of 1× TAE buffer, mix 5 mL of 10× TAE buffer stock solution in a bottle with 45 mL of Milli-Q water.
  b. Dissolve 0.3 g of agarose low EEO gel in 50 mL of 1× TAE buffer to prepare 0.6% agarose gel.
  c. Heat the agarose solution in the microwave for 1 min to solubilize, until the mixture becomes completely clear.
  d. Assemble the gel casting tray, while the agarose solution cools down.
  e. Once cooled, add 5 μL of gel red stain (nucleic acid stain) per 50 mL of agarose solution.
  f. Cast the gel, place the comb, and allow the gel to solidify in the gel tray.
  g. To prepare the samples, on a parafilm, mix the sample with 6× gel loading dye by pipetting up and down.
  h. Load the mixed sample into the gel (10 μL) after removing the comb.
  i. Keep one well for loading the GeneRuler 1 kb DNA ladder (4–5 μL).
  j. Cover the chamber and connect the apparatus to the power supply.
  k. Run the gel at 100 voltages for 30 min–1 h in 1× TAE running buffer.
  l. Visualize the gel using a Gel-doc machine (Figure 3B).
  m. Purify the PCR product using the Gene JET Gel Extraction Kit following the manufacturer’sinstructions.
    i. Visualize the gel on a UV transilluminator and excise the DNA band of interest, using a razor blade.
    ii. Place the excised gel slice in a pre-weighed microcentrifuge tube and reweigh to determine the weight of the gel slice.
    iii. Add binding buffer at 1:1 volume (μL): gel (mg) ratio and incubate on a heat block at 60°C to completely dissolve the gel.
    iv. Transfer up to 800 μL of the solubilized gel solution to the Gene-JET purification column.
    v. Centrifuge for 1 min at 12,000 × *g* and discard the flow through.
    vi. Add 700 μL of wash buffer to the column, re-centrifuge at the same speed, and discard the flow through.
    vii. Centrifuge the Gene-JET purification column for additional 1 min to remove residual wash buffer.
    viii. Transfer the GeneJET purification column into a clean 1.5 mL microcentrifuge tube.
    ix. Elute the purified DNA by adding 30–50 μL of warmed elution buffer at the center of the purification column. Incubate for 5 min at 22°C–25°C, then centrifuge at 12,000 × *g* for 2 min.
    x. Measure the concentration of the purified DNA using a Nano-Drop 2000c spectrophotometer.

**Pause point:** You can store the PCR amplicon at −20°C and proceed next day.

4. To generate compatible ends of RV-G amplicon and pVSV-dG-GFP-2.6, prepare two restriction digestion reactions with each of the pVSV-dG-GFP-2.6 vector and the RV-G amplicon in 0.2 mL PCR tubes as follows*:*

**Table:** 

Reagent	Amount (50 μL)
RV-G amplicon or pVSV-dG-GFP-2.6	500 ng
MluI-HF enzyme	1 μL
NheI-HF enzyme	1 μL
10× CutSmart Buffer	5 μL
NFW up to	50 μL

**CRITICAL:** Prepare several restriction digestion reactions and elute in small volumes (20 μL) to obtain high DNA concentration for the ligation reactions.

5. Incubate the restriction digestion reactions for 18 ± 2 h at 37°C.
6. Day 2: Heat inactivate the restriction digestion reaction at 80°C for 20 min. In the meantime, prepare the agarose gel electrophoresis to purify and confirm the digested pVSV-dG-GFP-2.6 and RV-G amplicon as described in step 3 (Figures 3C and 3D).
7. To ligate the digested RV-G amplicon into pVSV-dG-GFP-2.6, set up a ligation reaction in 0.2 mL PCR tubes as follows:

**Table:** 

Reagent	Amount (10 μL)
pVSV-dG-RV-G-GFP plasmid	100 ng/ μL
T4 DNA Ligase Buffer (10×)	1 μL
DNA insert (RV-G amplicon) (1.5 kb)	11.28 ng (1:1) or 33.3 ng (5:1)
T4 DNA Ligase	1 μL
NFW up to	10 μL

8. Incubate the ligation reaction for 18 ± 2 h at 16°C.
9. Day 3: Heat inactivate the ligation reaction at 65°C for 20 min.
10. Transform the ligation reaction (10 μL) into *DH5α E. coli* competent cells as described above (before you begin section, step 3).
11. During the incubation, prepare LB agar plates with selective bacterial medium by autoclaving the LB agar. Allow the solution to cool to 50°C–55°C (the ideal temperature for adding ampicillin at a final concentration of 100 μg/mL).
12. On a clean bench, pour the LB gar into petri dishes and allow the plates to solidify at 22°C–25°C.
13. After incubating the transformation mixture, plate 50 μL of the transformation mixture on the prepared LB agar plates and incubate for 18 ± 2 h at 37°C.

**CRITICAL:** Include control LB agar plates transformed with (1) cut vector only (to ensure colonies are not from vector recircularization), (2) uncut vector only (to confirm viability of competent cells).

14. Day 4: Check the plates for colonies. Pick single, well-defined colonies, and incubate each of them individually in 5 mL LB broth containing 100 μg/mL ampicillin for 18 ± 2 h, in a shaking incubator at 37°C. Troubleshooting 1.
15. Day 5: Use 4.5 mL of the bacterial culture for plasmid purification using Qiagen miniprep kit following the manufacturer’s instructions.

**CRITICAL:** Purification of pVSV-dG-RV-G-GFP vector, using midi prep kit, resulted in very low plasmid concentrations. Thus, we recommend purifying the pVSV-dG-RV-G-GFP using the mini-prep kit.

16. Mix the remaining culture (0.5 mL) with 50% glycerol stock in Milli-Q water and store at −80°C for future use.
17. To validate proper cloning of the RV-G insert into pVSV-dG-GFP-2.6, perform colony PCR, and diagnostic restriction digestion.
  a. Colony PCR.
    i. Transfer 1 mL of bacterial culture of each selected colony into separate 1.5 mL microcentrifuge tubes.
    ii. Centrifuge the bacterial cultures at 13,000 × *g* for 10 min, discard the supernatant and resuspend the pellet in 40 μL NFW with mixing.
    iii. Incubate the samples at 100°C for 10 min in bench incubator, then centrifuge at 13,000 × *g* for 5 min.
    iv. Transfer the DNA containing supernatant to a new microcentrifuge tube to serve as template DNA.
    v. Perform PCR using RV-G gene specific primers (RV-G-F and RV-G-R) and visualize the amplified region with agarose gel electrophoresis as described in steps 1–3 (Figure 3E).
  b. Diagnostic restriction digestion.
    i. Upon purification of plasmid DNA, set up a restriction digestion reaction as described in steps 4–6 (Figure 3F).

### Rescue of rVSV-dG-RV-G-GFP

**Timing: 6–7 days**

For the recovery of infectious virus from pVSV-dG-RV-G-GFP, co-transfect the modified pVSV-dG-RV-G-GFP along with VSV helper plasmids.^12^

18. Day 1: In two six well plates, seed BHK-21 cells at a density of 0.3 × 10^6^ cells/well in 1.5 mL growth medium, and incubate at 37°C /5% CO_2_ for 24 h.
19. Day 2: Once BHK-21 cells reach 80%–90% confluency, aspirate the growth medium and wash the cells once with 1× PBS. In the meantime, prepare the the rFPV virus inoculum (at MOI of 2), by mixing with 800 μL DMEM in 1.5 mL microcentrifuge tubes on ice.
20. Aspirate the PBS and inoculate the BHK-21 cells with the prepared rFPV inoculum as a source of T7 promoter.
21. Incubate the infected cells at 37°C for 2 h with shaking every 15–20 min, to allow uniform virus distribution.

**CRITICAL:** It is recommended to use rFPV instead of vaccinia virus as a source of T7 polymerase, since rFPV infection in mammalian cells does not produce infectious virus and unlike, vaccinia virus, rFPV does not interfere with infection studies involving other viruses.^13^

22. During incubation, prepare the transfection mixture with pVSV-dG-RV-G-FP and VSV helper plasmids in 15 mL conical tube at the following ratios:

**Table:** 

Plasmid	Concentration
pVSV-dG-RV-G-GFP	2.5 μg
pBS-N-ФT	0.75 μg
pBS-P-ФT	1.25 μg
pBS-L-ФT	0.5 μg
pBS-G-ФT	2.5 μg

23. Dilute the prepared plasmids in 750 μL Opti-MEM.
24. Mix the diluted DNA with 22.5 μL of turbofect transfection reagent (plasmid: turbofect ratio, 1 μg DNA plasmid: 3 μL turbofect).
25. Vortex the transfection mixture, then incubate it at 22°C–25°C for 25 min.

***Alternatives:*** Other transfection reagents can be used such as lipofectamine or viafect.

26. After 2 h, remove the rFPV inoculum, and wash the infected cells 3 times with 1× PBS.
27. Remove the 1× PBS and add the transfection mixture in a dropwise manner to the cells with swirling the plates gently.
28. Incubate the plates at 37°C/ 5% CO_2_ for 16 ± 2 h.

**CRITICAL:** Include control wells as follows: (1) Wells infected with rFPV, then transfected with pCITE-GFP plasmid control (encodes the ORF of GFP under the control of T7 promoter), to ensure the efficiency of T7 promoter in rFPV virus. (2) Wells transfected with pCAGG-GFP only. (3) Wells infected with rFPV, then transfected with pVSV-dG-GFP-2.6 and VSV helper plasmids.

***Note:*** It is recommended to prepare 8 transfected wells with the prepared plasmids mixture to obtain sufficient volumes of the rescue virus.

29. Check the GFP daily using fluorescence microscope and examine the cytopathic effect (CPE) evidenced by syncytia formation.

**Figure 4:**
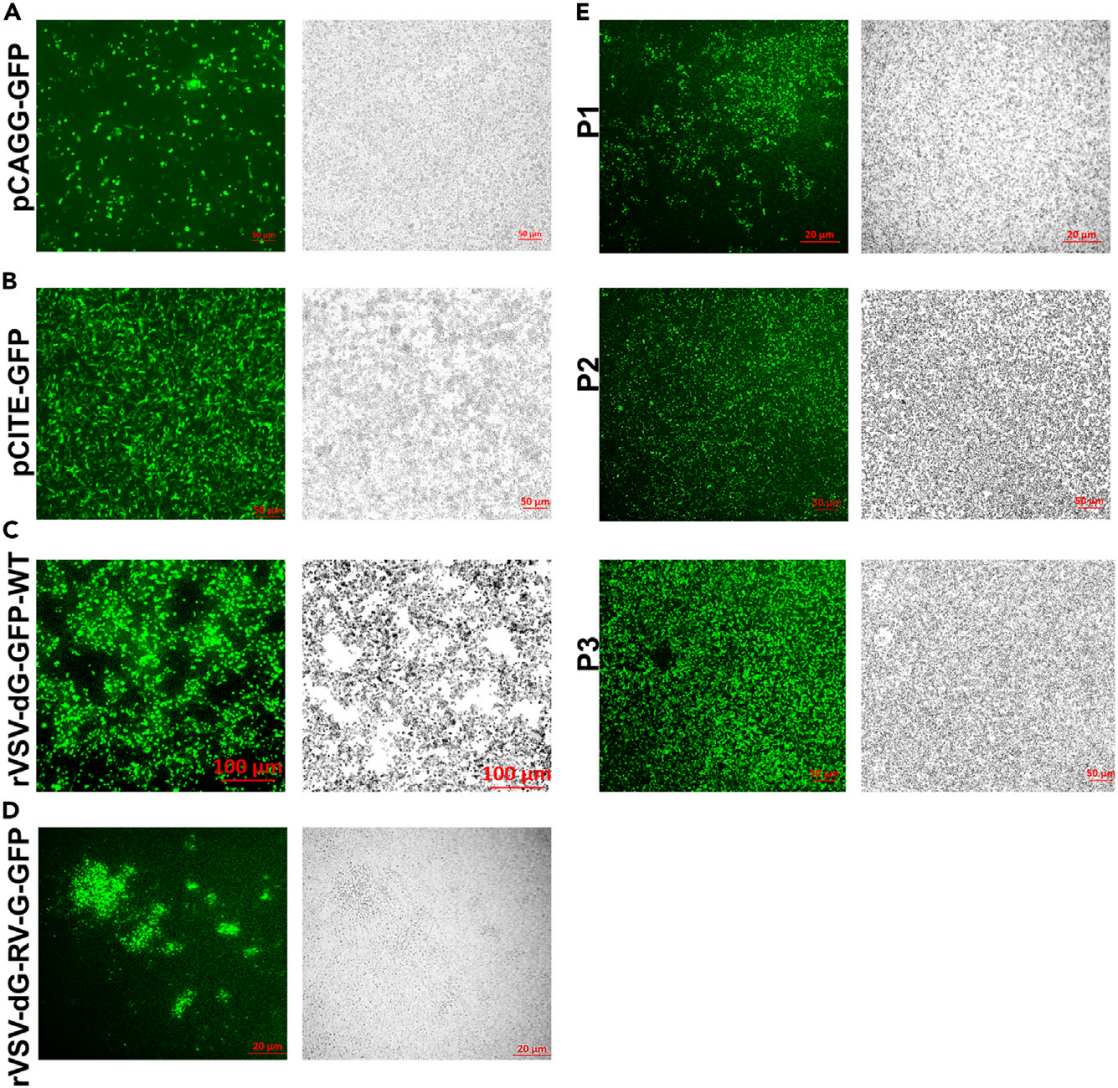
Transfection and rFPV-T7 infection efficiencies (A) Representative microscopic fluorescent (left) and bright (right) fields of BHK-21 cells, transfected with pCAGG-GFP, illustrated as transfection control. Scale bars size: 50 μm. (B) Representative microscopic fluorescent (left) and bright (right) fields of BHK-21 cells infected with rFPV-T7 and transfected with pCITE-GFP, illustrated as transfection control. Scale bars size: 50 μm. (C) Representative microscopic fluorescent (left) and bright (right) fields of the BHK-21 cells, 24 h post transfection with pVSV-dG-GFP-2.6 and VSV helper plasmids. Scale bars size: 100 μm. (D) Representative microscopic fluorescent (left) and bright (right) of the BHK-21 cells, 72 h post transfection with rVSV-dG-RV-G-GFP along with VSV helper plasmids (P0). Scale bars size: 20 μm. (E) Microscopic fluorescent (left) and bright (right) of the BHK-21 cells cytopathic effects appeared 72 h following the inoculation of the BHK-21 cells with the subsequent passages of the recovered VSV-dG-RV-G-GFP. Images representing the subsequent passages of rVSV-dG-RV-G-GFP; P1, P2 and P3. Scale bars size: 20 and 50 μm.

***Note:*** Twenty-four hours post transfection, GFP is observed in the following wells: wells transfected with pCAGG GFP only, wells infected with rFPV and transfected with pCITE GFP, wells infected with rFPV and transfected with pVSV-dG-GFP-2.6 along with VSV helper plasmids (Figures 4A–4C).

30. After 72 h, the GFP is observed in wells infected with rFPV and transfected with pVSV-dG-RV-G-GFP. Once GFP is observed, collect the virus by freezing and thawing method:
  a. Virus harvest:
    i. Collect the cell culture containing infected cells.
    ii. Transfer the cell suspension into 15 mL falcon tube.
  b. First freezing cycle:
    i. Freeze the samples at −80°C.
    ii. Allow the samples to freeze completely. Typically, this can take around 30 min to a few hours.
  c. First thawing cycle:
    i. Thaw the samples rapidly by placing them in a 37°C incubator.
    ii. Gently agitate the samples during thawing to ensure even warming and to avoid temperature gradients within the sample.
  d. Repeat the freezing and thawing cycles for 2 times.
  e. After the final thawing, centrifuge the recovered virus at 300 × *g* for 5–10 min to pellet cell debris.
  f. Carefully collect the supernatant containing the released viral particles in sterile conical tubes, then pass the virus through 0.22 μm syringe filter to remove the rFPV.
31. Aliquot the recovered virus in sterile microcentrifuge tubes and keep at −80°C for future use (Figure 4D). Troubleshooting 2.
32. Inoculate the rescued VSV-dG-RV-G-GFP on BHK-21 cells to ensure the stability of the RV-G insert and carry out further passaging (Figure 4E). Troubleshooting 3.

### Molecular characterization of rVSV-dG-RV-G-GFP

#### Reverse-transcription polymerase chain reaction (RT-PCR)

**Timing: 2 days**

To confirm the stability of RV-G insert in the rVSV-dG-RV-G-GFP subsequent passages, carry out RT-PCR through the following steps.

33. Day 1: Viral RNA extraction.
  a. Extract the viral RNA from the following wells: (1) viral supernatant of rVSV-dG-RV-G-GFP 3^rd^ passage and (2) viral supernatant of rVSV-GFP-WT, using QIAamp Viral RNA Mini Kit (QIAGEN) kit following the manufacturer’s instructions.
  b. In 1.5 mL sterile, clearly labeled microcentrifuge tubes, add for each 140 μL viral supernatant, 560 μL lysis buffer AVL and 5.6 μL carrier RNA, mix with pulse vortexing for 15 s.
  c. Incubate the mixture at 22°C–25°C for 10 min and briefly centrifuge to remove drops from the inside lid.
  d. Add 560 μL absolute ethanol to the mixture and mix by pulse-vortexing, then briefly centrifuge to remove drops from the lid.
  e. Label and prepare the QIAamp Mini column for each sample, transfer 630 μL of the lysate into the QIAamp Mini column, then centrifuge at 6000 × *g* for 1 min.
  f. Discard the collection tube and place the column in a new collection tube.
  g. Repeat the previous step until the samples are fully loaded.
  h. Wash the column twice as follows: once with 500 μL AW1 Buffer and the next wash with 500 μL of Buffer AW2.
  i. After each wash, centrifuge the column at 6000 × *g* for 1 min in the initial wash and for 3 min at 20,000 × *g* in the second wash, then place into clean collection tube.
  j. Centrifuge the spin column for 1 min at full speed for removal of any residual washing buffer.
  k. To elute the viral RNA, add a volume of 30 μL AVE buffer to the center of the membrane after setting in a sterile, labeled microfuge tube then incubate for 5 min at 22°C–25°C
  l. After incubation, centrifuge the tubes at 6000 × *g* for 1 min.
  m. Quantify the extracted viral RNA using nanodrop spectrophotometer with checking and recording the RNA quality.

**Pause point:** The extracted viral RNA can be stored at −80°C before further processing.

34. Day 2: Set up the RT-PCR reaction.
  a. The extracted viral RNA is used as a template for RT-PCR. Prepare the cDNA synthesis mixture in 0.2 mL thin-walled PCR tube for each sample.
  b. Prepare a 20 μL reaction with the following components in two steps:
    i. Set up the following reaction mixture on ice to anneal the primer to template RNA (step 1):

**Table:** 

Component	Volume
2 μM gene-specific reverse primer	1 μL
10 mM dNTP mix (10 mM each)	1 μL
Template RNA (100 ng RNA)	8 μL
DEPC-treated water	Up to 13 μL

      **CRITICAL:** Mix the reaction components, then heat the template RNA primer mixture at 65°C for 5 min, then incubate on ice for 1 min.
    ii. Set up the RT reaction mixture (step 2) as follows:

**Table:** 

Component	Volume
5× SSIV Buffer	4 μL
100 mM DTT	1 μL
RNase-OUT Recombinant RNase Inhibitor	1 μL
Super-Script IV Reverse Transcriptase (200 U/ μL)	1 μL
    iii. Mix the contents of both reactions and incubate at 55°C for 10 min.
    iv. Incubate the reaction at 80°C for 10 min to inactivate it.
    v. Prepare the PCR reaction using VSV-up and VSV down primers as demonstrated in the schematic diagram (Figure 5A).

**Table:** 

Component	Amount (25 μL)	Final concentration
DNA template	1 μL	100 ng
Dream-Taq Green PCR Master Mix (2×)	12.5 μL	0.02 U/μL
10 mM VSV-UP primer	0.625 μL	0.5 μM
10 mM VSV-Down primer	0.625 μL	0.5 μM
NFW	up to 25 μL	–
    vi. Set up the PCR thermocycler at the following conditions:

**Table:** 

Steps	Temperature	Time	Cycles
Initial Denaturation	95°C	3 min	1
Denaturation	95°C	30 s	35 cycles
Annealing	67.8°C	1 min	
Extension	68°C	2 min	
Final extension	68°C	10 min	1
Final Hold	4°C	–
    vii. Upon amplification, perform agarose gel electrophoresis (Figure 5B).

**Figure 5:**
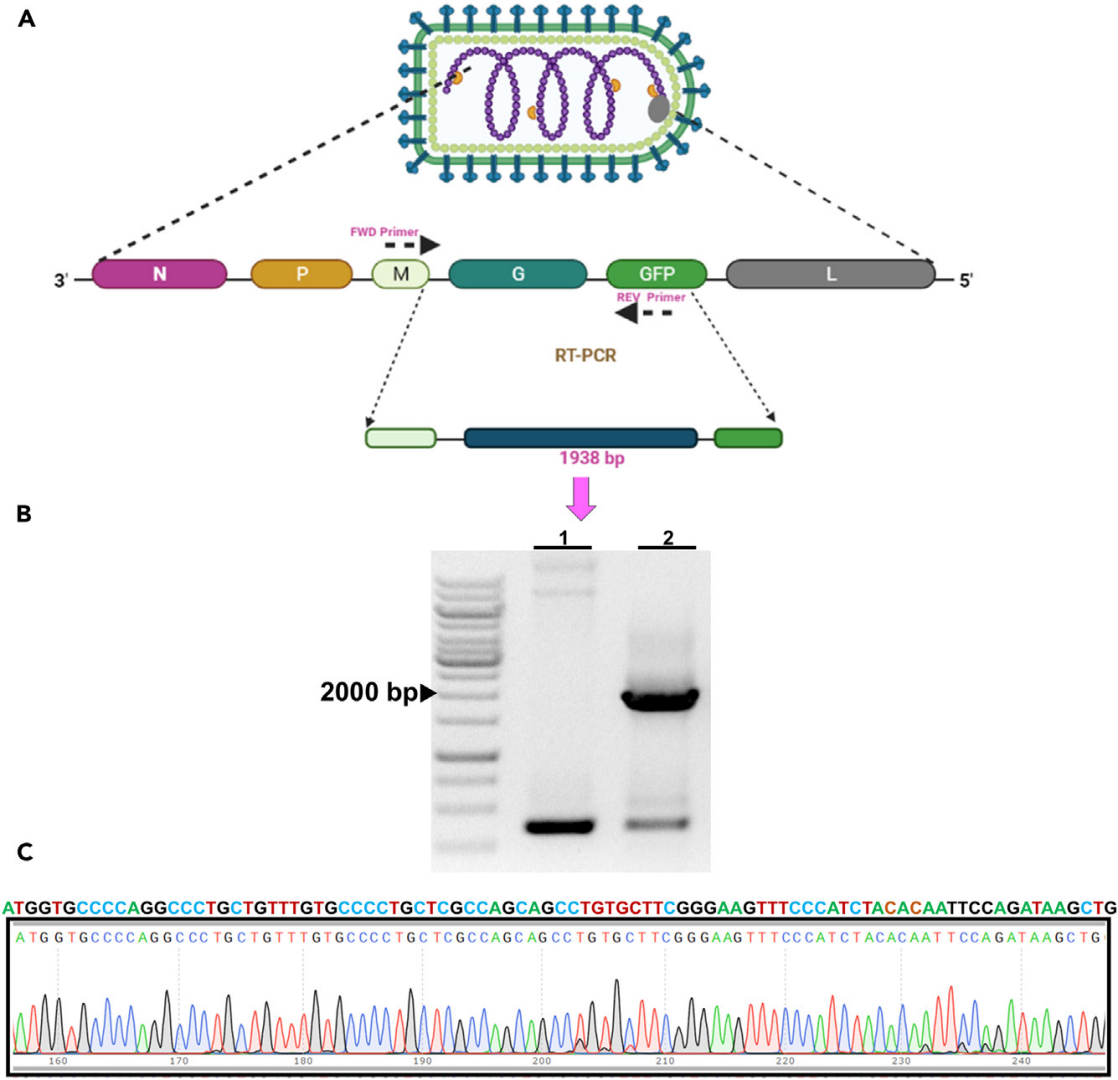
Genetic stability of RV-G insert in rVSV-dG-RV-G-GFP (A) Schematic diagram of the rVSV-dG-RV-G-GFP viral genome organization: 3′-N, P, M, G, GFP and L-5′, with FWD (VSV-Up) and REV (VSV down) primers designed to target the DNA vector, flanking the RV-G insert. (B) Agarose gel electrophoresis showing RT-PCR analysis of RNA extracted from (1); rVSV-GFP-WT and (2); rVSV-dG-RV-G-GFP using VSV UP and VSV down primers, DNA ladder (1 kb). (C) Forward Sanger sequencing of the RV-G amplicon, amplified from rVSV-dG-RV-G-GFP using VSV up primer, showing the start codon of RV-G with no deletions or insertions in the RV-G-gene.

### Sanger sequencing

**Timing: 2–3 days**

35. To verify the stability of RV-G insert in the rescued rVSV-dG-RV-G-GFP, prepare the concentration of primers (VSV-UP and VSV-down primers) and plasmids according to the sequencing company specifications.
36. Analyze the sequence contigs using the NCBI BLAST tool (Figure 5C).

### Assessment of rVSV-dG-RV-G-GFP replication fitness

#### Plaque assay

**Timing: 5–6 days**

Plaque assay represents a quantitative method for measuring the infectious virus progeny through plaques quantification of culture infected cells with serial dilution of the virus specimens.^14^ While viral RNA quantification provides highly specific and sensitive method, it does not usually reflect whether all the replicating viral genome copies could release infectious virus particles. Herein, we explain the plaque assay utilizing liquid overlay medium.

37. Day 1: In a 6 well plate, seed BHK-21 cells at a density of 0.3 × 10^6^ cells/well. Incubate the plate at 37°C with 5% CO_2_, until reaching 70–80% confluency.
38. On the same day, prepare and autoclave 3% CMC solution. Allow the solution to cool to 22°–25° C, then store it for 18 ± 2 h at 4°C to ensure complete solubilization.
39. Day 2: In the morning, transfer the 3% CMC solution bottle from 4°C to the bath bead and set the temperature at 37°C to warm the solution.
40. In the bio safety cabinet, perform ten-fold serial dilution of the virus supernatants (rVSV-dG-RV-G-GFP and rVSV-GFP-WT) by adding 900 μL of infection medium to each 1.5 mL tube. Transfer 100 μL of each lower dilution specimen to the subsequent tube to obtain higher dilution.
41. Prepare the virus supernatant dilutions as follows: 10^−1^, 10^−2^, 10^−3^, 10^−4^, 10^−5^, and 10^−6^, Vortex each dilution for proper mixing.

***Note:*** If the virus titer is too high, perform until the 10^th^ dilution.

**CRITICAL:** Perform the specimens’ dilutions in triplicates and for each plate, set uninfected wells to serve as negative control. Properly label the six well plates with the corresponding dilutions.

42. After preparation of the serially diluted virus, aspirate the growth medium from BHK-21 cells and wash the cells once with 1× PBS.
43. Aspirate the PBS, then add 900 μL of the prepared virus dilution designated for each well after vortexing each tube.
44. Incubate the plates at 37°C in a 5% CO_2_ incubator for 2 h, with shaking every 15–20 min.
45. During virus incubation, prepare the overlay medium and mix it with equal volume of 3% CMC solution to use as the plaque overlay medium.

**CRITICAL:** Keep the prepared overlay medium on a magnetic stirrer until use.

46. After 2 h of virus infection, transfer the plates from the incubator and place them in the biosafety cabinet, discard the diluted virus supernatants, then wash the infected cells 3 times with 1× PBS.
47. Aspirate, the PBS, then slowly add 4 mL of the prepared plaque overlay medium/ well using 25 mL stripettor.

***Note:*** Start from uninfected wells to wells infected with highest virus dilution, then finally to wells with the lowest virus dilutions.

48. Carefully, transfer the plates into 37°C incubator with 5% CO_2_ for 72 h.
49. Check the plates daily for plaque formation and for GFP.
50. After 72 h, transfer the plates from the incubator into biosafety cabinet class II, add 1 mL of 4% PFA for each well for to fix the cells. Begin with wells of uninfected cells, followed by wells of highest virus dilutions then lowest virus dilutions.
51. Place the plates on a shaker at 22°C–25°C for 1 h.
52. After 1 h, tilt the plates to your side and, gently aspirate the overlay medium from the plates using a stripettor.

**CRITICAL:** Discard the overlay medium in a waste bottle with detergent.

**CRITICAL:** Ensure not to disrupt the cell monolayer to maintain the rounded shape of formed plaques.

53. Upon removal of the overlay medium, stain the cells by adding 1 mL of 0.5% crystal violet and keep on a shaker for 1 h at 22°C–25°C.
54. To count the plaques, remove the stain and wash the wells gently with water until the rounded plaques appear in a purple monolayer. Troubleshooting 4.

**Figure 6:**
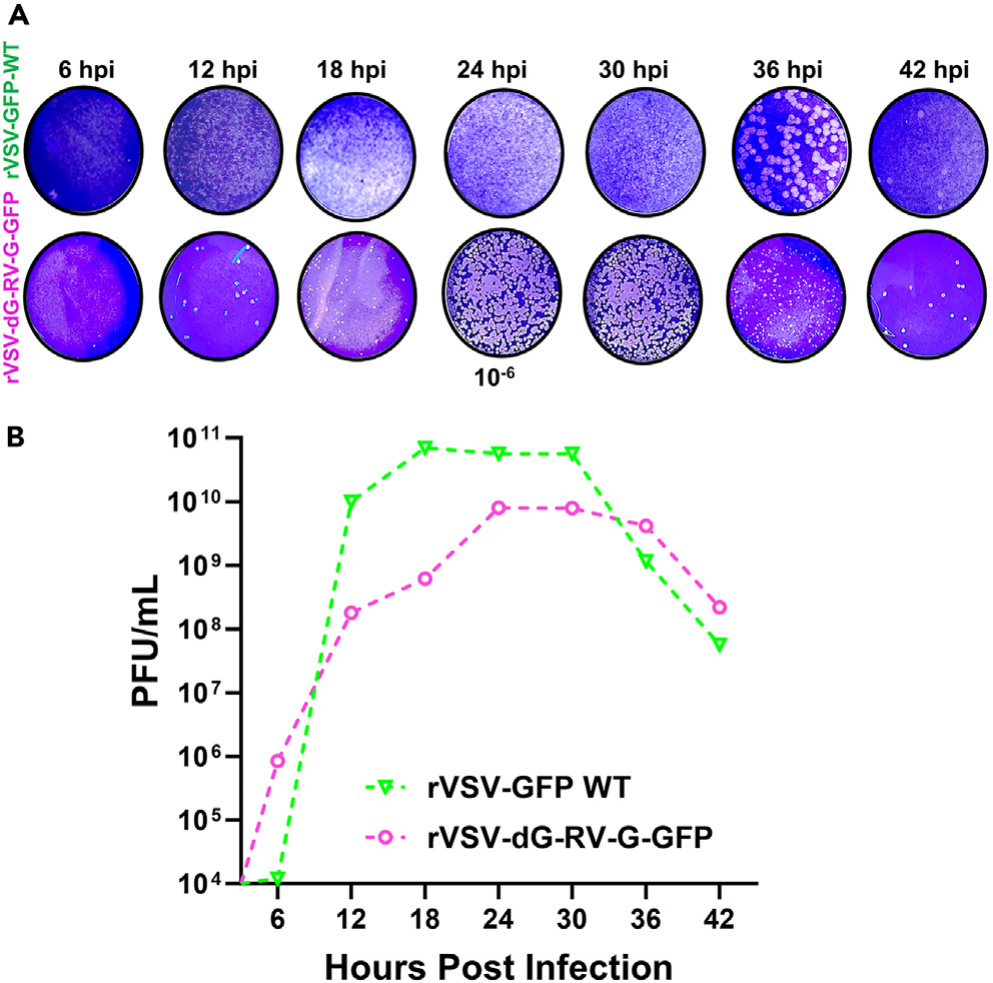
Plaque assay of rVSV-dG-RV-G-GFP and rVSV-GFP-WT in a time dependent manner (A) Representative plaque counts of BHK-21 infected cells with either rVSV-GFP-WT or rVSV-dG-RV-G-GFP at MOI = 1 at the 6^th^ virus dilution in the indicated time points, with plaques stained using 0.5% crystal violet, 72 hpi. These experiments were performed three times independently (*n* = 3). (B) Plaque assay-based quantifications of the progeny virus released from the BHK-21 cells infected with either rVSV-GFP-WT or rVSV-dG-RV-G-GFP at MOI = 1, with viral supernatants collected in a time-dependent manner (6 hpi −42 hpi). The data represented the mean of three biological replicates and the error bars represent the SEM.

***Note:*** The uninfected reference control should appear as uniform monolayer without any plaques (Figures 6A and 6B). Count the plaques at the highest virus dilution with 5–100 plaques using the following equation: Virus titer (PFU/mL) = Average number of plaques given by the identified virus dilution/ (dilution factor × volume of inoculum per plate).

**CRITICAL:** Dispose the plates in biohazardous waste, Incubate the liquid waste with detergent at 22°C–25°C for 18 ± 2 h before disposal.

#### Quantitative one step RT-qPCR (TaqMan)

**Timing: 5–6 days**

55. Day 1–4: Viral RNA extraction.

Extract the viral RNA of infected BHK-21 cells with either rVSV-dG-RV-G-GFP or rVSV-GFP-WT as described above in step 33 (see molecular characterization, RT-PCR).

56. Day 5: Standard curve.
  a. Upon extraction of the viral RNA, prepare different viral RNA concentrations and quantify the genomic viral RNA in each of the prepared samples, using the SuperScript III Platinum One-Step RT-qPCR Kit following the manufacturer’s instructions.
  b. Set up the RT-qPCR reactions on ice, prepare a master mix, followed by adding the reactions in plate wells on ice.
    **CRITICAL:** Add the template viral RNA at the end separately to each well.
    ***Note:*** Each of the viral RNA concentrations is performed in triplicates.
  c. Set up the One-step RT-qPCR reaction in 96-well PCR plates as follows.

**Table:** 

Component	Volume (50 μL)	Final concentration
SuperScriptIII RT/Platinum *Taq Mix*	1 μL	–
2× Reaction Mix	25 μL	–
FWD primer	1 μL	10 μM
REV primer	1 μL	10 μM
Fluorogenic probe	0.5 μL	10 μM
RNaseOUT	1 μL	–
RNA template	10 μL	1 pg to 1 μg
DEPC-treated water	Up to 50 μL	–
  d. Upon setting up the reactions, seal the 96 well plate with micro seal adhesive film.
  e. Briefly, centrifuge the plates to collect plate contents.
  f. Place the plate in CFX96 Real-Time system and set up the RT-qPCR profile of the quantitative One step RT-qPCR (TaqMan) as follows:

**Table:** 

Temperature	Duration	No of cycles
50°C	15 min	–
95°C	2 min	–
95°C	15 s	40
60°C	30 s	

**Figure 7:**
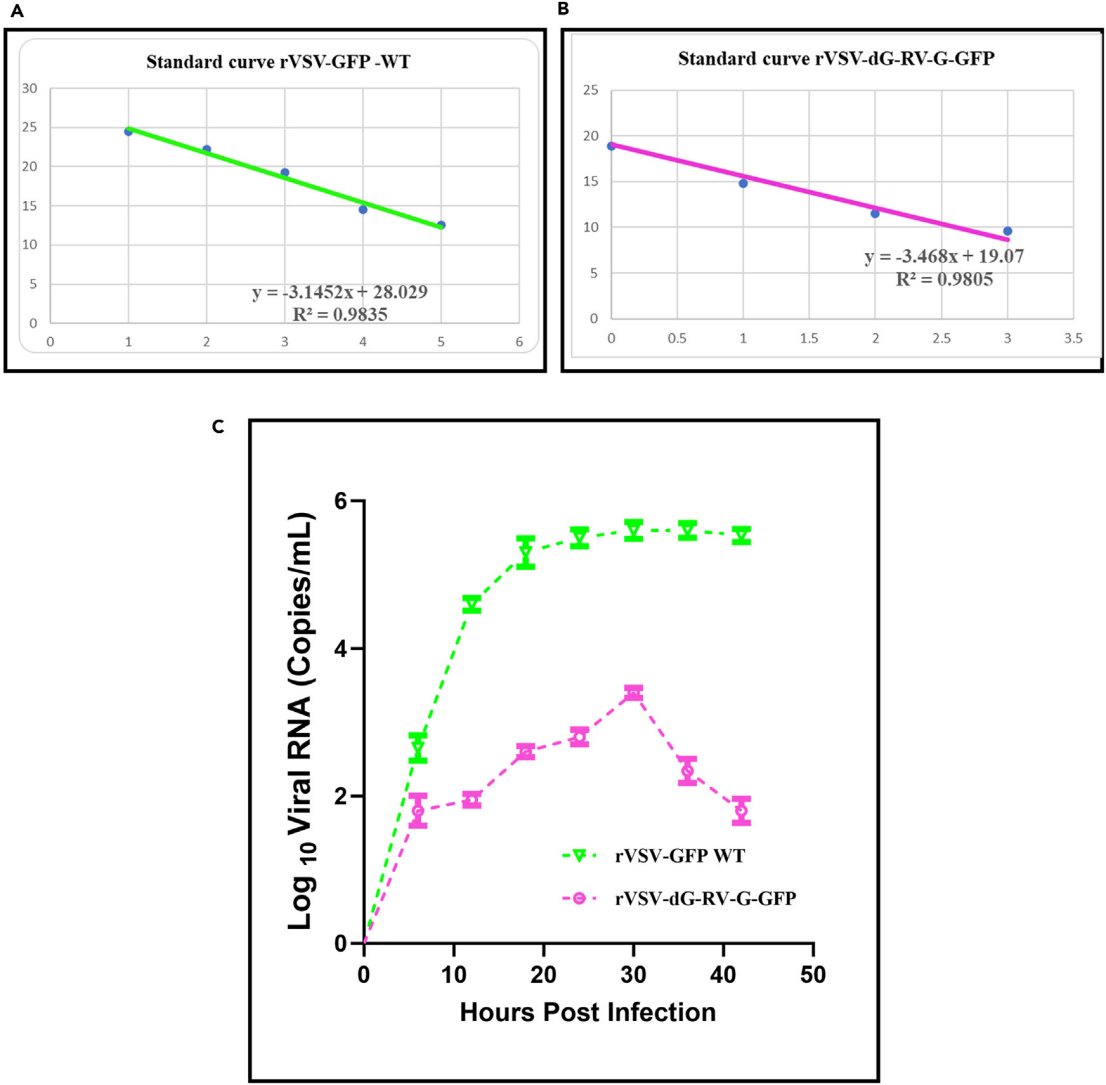
Growth kinetics of rVSV-GFP-WT and rVSV-dG-RV-G-GFP infected BHK-21 cells quantified by qRT-PCR at multiple time points post infection (A) Standard curve of rVSV-GFP-WT viral RNA generated using ten-fold dilutions (0.1–1000 ng), the X-axis represents the RNA quantity and Y-axis shows CT values. (B) Standard curve of rVSV-dG-RV-G-GFP viral RNA, generated using ten-fold dilutions (0.1–1000 ng), plotted as X-axis and the CT values are indicated on Y-axis. (C) Absolute quantification of the viral RNA copies on BHK-21 cells, infected with either rVSV-GFP-WT or rVSV-dG-RV-G-GFP at MOI = 1. Viral supernatants were collected at different time points post infection and assessed for the absolute quantities of the genomic RNA by qRT-PCR targeting the VSV N gene. The data represent the mean values of three biological replicates. Error bars represent the SEM.

Melt curve 65°C–95°C increment 0.5 for 0.05 +plate read.
  g. Once the cycle ends, copy the files and analyze the data.
  h. In Excel file, generate two standard curves for each of rVSV-dG-RV-G-GFP and rVSV-GFP-WT, representing the log of copy number values (different dilutions) on the *X*-axis versus the CT values obtained for each of the viral RNA dilutions presented on the *Y*- axis (Figures 7A and 7B).
  i. From the plotted standard curve in Excel sheet, right click on the graph, select trendline option, click display equation then an equation appears as follows:
    Y,CTvalues=(−3.1452(m),slope)X,logquantity+21.74(b),intercept.
  j. To calculate the efficiency of qPCR reaction, use the following equation:
    **Efficiency; e = (10**^**(-1/m;slope)**^**-1)** × **100**, efficiency could also be calculated from this website by entering the slope. where: e = theoretical efficiency, Slope = the slope of the standard curve, plotted with the y axis as Ct and the x axis as log(quantity). For rVSV-dG-RV-G-GFP and rVSV-GFP-WT, the efficiency of qPCR was 94.24 and 107%; respectively.
    ***Note:*** Typically, the amplification efficiency ranges between 90-110%.
  k. To quantify the genomic viral RNA copies of rVSV-dG-RV-G-GFP and rVSV-GFP-WT in time dependent manner:
    i. Infect BHK-21 cells (70–80% confluency) with rVSV-dG-RV-G-GFP or rVSV-GFP-WT at MOI = 1.
    ii. Collect the viral supernatants at different time intervals from 6 h until 42 h post infection (hpi), to assess the replication of both viruses and to determine the peak of virus replication.
    iii. Extract the viral RNA from the collected viral supernatants and set up qRT-PCR reaction for all obtained specimens as described above.
    iv. After obtaining the CT values of the samples, calculate their mean and standard error at the different time points.
    v. Quantify viral RNA copies of rVSV-dG-RV-G-GFP and rVSV-GFP-WT at the different time points using the following equation:
      $$T\;\left(\text{quantity}\right)=10^{(\text{CT}\;\left(\text{Cycle}\;\text{Threshold}\right)-b\left(\text{intercept}\right)/m\left(\text{slope}\right)}$$
    vi. Then calculate Log _10_ of the obtained values and plot as growth curves in GraphPad Prism (Figure 7C).
    vii. As an example, from the generated standard curve, we obtained a slope (m) of −3.14 and an intercept (b) of 28.03. The average CT value obtained for the sample was 19.68. The quantity(T) of N gene in the sample is calculated using the following equation:
      $$T\;\left(\text{quantity}\right)=10^{(\text{CT}\;\left(\text{Cycle}\;\text{Threshold}\right)-b\left(\text{intercept}\right)/m\left(\text{slope}\right)}\text{.}$$
      T = 10^(19.68-28.03)/-3.14^ = 456.3. then calculate log _10_ the obtained value, log _10_ = 2.66.

### Structural characterization

#### Immunofluorescence staining assay

**Timing: 4–5 days**

To demonstrate the localization of VSV-M and RV-G proteins in each of rVSV-dG-RV-G-GFP and rVSV-dG-GFP-WT, perform IFA assay^15^ as follows.

57. Day 1: In a 24 well plate, seed BHK-21 cells at a density of 5 × 10^4^ cells/well on cover slips, incubate the plate at 37°C/5% CO_2_ for the next day.
58. Next day, upon reaching 70–80% cell confluency, wash the cells with 1× PBS.
59. While washing the cells, prepare the virus inoculum of each of rVSV-dG-RV-G-GFP and rVSV-dG-GFP-WT by diluting in infection medium (MOI of 1).
60. Discard the PBS from BHK-21 cells, then inoculate the prepared virus onto the cells. Incubate the plates at 37°C for 2 h with shaking.
61. Two hrs post infection, remove the virus inoculum and wash the cells 3× with 1× PBS, then aspirate the PBS and add 0.5–1 mL of growth medium.
62. Day 3: Check the cells for GFP to ensure successful infection and wash the infected cells three times with 200 μL/well 1× PBS.
63. Aspirate the PBS, then add 200 μL /well of 4% PFA at 22°C–25°C for each well. After 1 h, remove the PFA and wash the cells with 1× PBS.

**CRITICAL:** Ensure proper rinsing after each step to avoid false positive results. Also, it is recommended to place the plate on a shaker for uniform distribution.

64. Aspirate the PBS, then add 200 μL/well of 0.1% Triton X-100 for 10 min to permeabilize the cells.
65. After 9 min, remove the triton and wash the cells 3 times with 1× PBS, allow 5 min/wash to remove all traces of the Triton solution.

**CRITICAL:** It is recommended to use triton X-100 as it permeabilizes the lipid bilayer and nuclear membrane. Do not incubate the triton with cells for longer times and avoid using high concentrations as it might destroy the cell membrane.

66. Aspirate the PBS, then add 200 μL/well of blocking buffer for 1 h at 22°C–25°C on a shaker, to block non-specific binding.
67. After 1 h, remove the blocking solution, and prepare the primary antibody targeting either the VSV-M or RV-G proteins as follows:
  a. Prepare VSV-M or RV-G antibody solutions at concentration of 1:400 for the primary antibodies.
    i. Dilute 3 μL of the primary antibody in 1200 μL 0.5% BSA solution.
    ii. Add 200 μL of the antibody solution / each well.
    iii. Incubate the plate with the primary antibody, for 18 ± 2 h at 4°C on a shaker.
    iv. Cover the plate with aluminum foil to prevent evaporation of the contents.
68. Day 4: Remove the primary antibody solution. Rinse the cells for 3 times with 1× PBS, 5 min/wash to remove excess antibody solution.
69. Prepare the secondary antibody solution as follows:
  a. Prepare a concentration of 1:800 for the secondary antibody solutions.
    i. Dilute 1.5 μL of the secondary antibody goat anti-mouse IgG Alexa-Fluor 488 in 1200 μL 0.5% BSA.
    ii. Add 200 μL of the antibody solution / each well.
    iii. Incubate for 1–2 h at 22°C–25°C on shaker and cover the plate with aluminum foil.
70. After 2 h, aspirate the secondary antibody solution, wash the cells with 1× PBS for 3 times, 5 min/wash, and do a final wash with distilled water then aspirate the distilled water.
71. For nuclei staining, add 200 μL of 4′,6-diamidino-2-phenylindole (DAPI), at a concentration of (1:10,000).

***Note:*** After DAPI staining**,** incubate the plates on a shaker for 30 min, cover the plates with aluminum foil.

72. During incubation, prepare glass slides mounted with 1 drop of VECTASHIELD (mounting medium). For each glass slide, mount 2 coverslips and clearly label them with the corresponding samples.
73. Gently dislodge the coverslips from the plates using forceps, then dip the coverslips in Milli-Q water.

***Note:*** Gently dry the coverslips’ edges in fiber free paper.

**CRITICAL:** Carefully, invert the cover slides, so that the side with cultured cells is in the bottom. To avoid any air bubbles, gently press the coverslip with the tip of a 1 μL pipette tip.

74. Add one drop of clear nail polish on each of the coverslip corners for sealing.

**CRITICAL:** Avoid dissemination of nail polish as that will interfere with visualization. Also, to ensure dryness of the nail polish, keep the microscopic slides for around 30 min to dry, in a dark place.

75. Store the slides at 4°C, and keep them covered, to be away from light, until imaging.

***Note:*** It is recommended to image the slides within 2 weeks, since prolonged storage of the slides might result in dryness of the cells.

**Figure 8:**
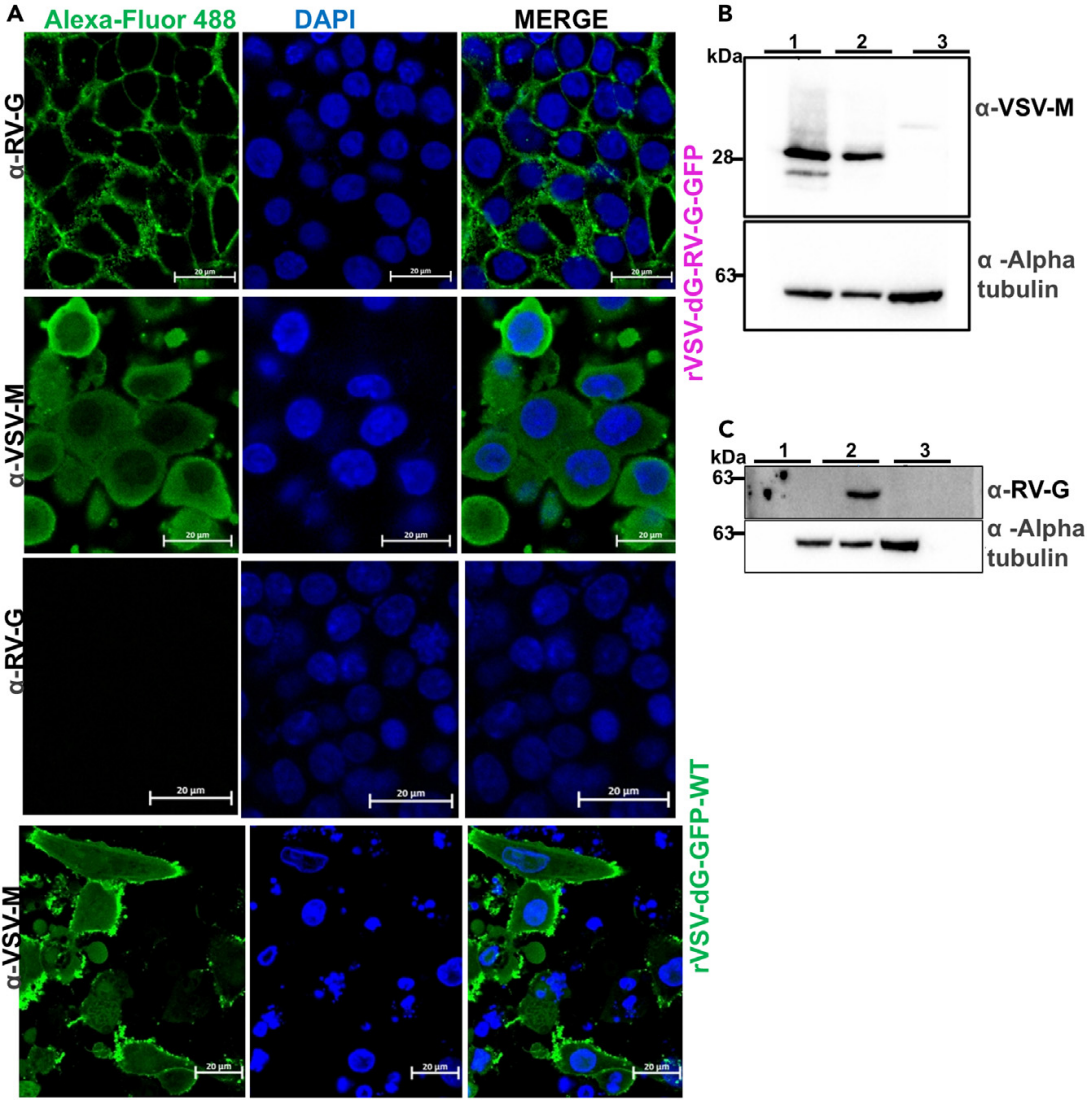
RV-G and VSV M proteins expression in BHK-21 cells infected with either rVSV-dG-RV-G-GFP or rVSV-dG-GFP-WT, analyzed by immunofluorescence and immunoblotting (A) Immunofluorescence microscopy showing surface expression of RV-G in rVSV-dG-RV-G-GFP infected cells and intracellular localization of VSV M observed in infected BHK-21 cells infected with either rVSV-dG-RV-G-GFP or rVSV-dG-GFP-WT. Nucleus (blue) labeled with DAPI, the VSV-M and RV-G (green), Scale bars are 20 μm. Fluorescence signals were visualized by confocal immunofluorescence microscopy. Images analyzed using the ZenCore 3.4 software. (B) Immunoblot analysis, showing VSV M protein expression in both (1) rVSV-dG-GFP-WT and (2) rVSV-dG-RV-G-GFP infected BHK-21 cell lysates, with alpha tubulin used as a loading control. (C) Immunoblot analysis, demonstrating RV-G protein expression in (2) rVSV-dG-RV-G-GFP infected BHK-21 cell lysates, but not in (1) rVSV-dG-GFP-WT infected BHK-21 cell lysates or (3) non infected cell lysates. Alpha tubulin was used as a loading control. The experiments were performed two times independently (*n* = 2). Images were analyzed using Image Lab software. Images of uncropped blots are shown in Figure S1.

76. Acquire the images with laser confocal microscope (LSM880). Execute and analyze the images using Zeiss software (Figure 8A).

### Western blot

**Timing: 6 days**

To confirm in frame cloning and expression of RV-G and to demonstrate the proper assembly of rVSV-dG-RV-G-GFP and rVSV-dG-GFP-WT, we assessed the expression of RV-G and VSV-M proteins by western blot.^16^

77. Samples preparation.
  a. Day 1: In two six well plates, seed BHK-21 cells, at a density of 0.3 × 10^6^ cells/well. Incubate the plates at 37°C/ 5% CO_2_ until the following day.
  b. Day 2: Upon reaching the cells 70–80% confluency, aspirate the growth medium and wash the cells once with 1× PBS.
  c. Infect 3 wells with rVSV-dG-RV-G-GFP at MOI = 1 and infect another 3 wells with rVSV-dG-GFP WT at MOI = 1. Keep 3 wells as mock control.
    i. Prepare the infection medium by diluting either the rVSV-dG-RV-G-GFP or rVSV-dG-GFP-WT in 900 μL DMEM only without serum or antibiotic and infect the cells with the inoculum.
    ii. Incubate the plates for 2 h at 37°C/5% CO_2_, with shaking every 15–20 min to allow even distribution of the virus into cells.
    iii. After 2 h, remove the inoculum and wash the cells 3 times with 1× PBS, then add the growth medium.
    iv. Day 5: Twenty-four hpi, wash the cells 3 times with 1× cold PBS, then add 200 μL of ice cold 1× PBS, scrape the cells using 1 mL pipette tip to detach the cells.
      **CRITICAL:** BHK-21 cell detachment is easily carried out by ice cold 1× PBS. For other adherent cell lines, gentle dissociation reagents such as Accutase could be used.
      ***Note:*** Preferably, from this step forward, perform all subsequent sample preparation steps on ice.
  d. Collect the PBS scraped cells into the corresponding labeled 1.5 mL labeled microcentrifuge tubes.
  e. Centrifuge the cells at 300 × *g* for 5 min, discard the supernatant and resuspend the cell pellet in 100 μL of 1% NP-40 lysis buffer (with protease inhibitor tablet) to obtain the whole cell lysates.
  f. Place the samples on ice and keep on a shaker for 30 min.
  g. Centrifuge the samples at 13000 × *g* for 20 min at 37°C.
  h. Discard the pellet and transfer the supernatant into fresh new, labeled 1.5 mL microcentrifuge tubes.
    **Pause point:** You can store the cell lysates at −80°C and proceed the following day.
78. Gel casting and preparation.
  a. Assemble the casting stand, casting frame, small and large glass plates 1.5 mm for gel casting as follows:
    i. Initially place the large rectangular casting stand on a clean, dry surface.
    ii. Align the bottom of small and large 1.5 mm glass spacers together, with the smaller plates facing the outside.
    iii. Secure the aligned glass plates into the frame by pushing gates of the frame out to the sides.
    iv. Assemble the casting frame with the glass plates into the casting stand.

**CRITICAL:** Check whether assembled plates in the casting stand are properly sealed, by pipetting 1 mL of water into the space between the plates, if no leaking, then it is ready to prepare the gels, pour the water added into a sink and dry the plates from any residual water. If leakage is observed, reassemble the plates in the casting frame within the casting stand.

For SDS-PAGE, resolving and stacking gels are required. a resolving gel in which proteins are resolved based on their molecular weights and a stacking gel in which proteins are concentrated before entering the resolving gel. Prepare the resolving and stacking gels as follows.

**Table:** 

Component	Resolving gel 12% (10 mL)	Resolving gel 8% (10 mL)	Stacking gel (4%) (4 mL)
Milli-Q water	3.3 mL	4.6 mL	2.7 mL
30% acrylamide-Bis acrylamide	4 mL	2.7 mL	670 μL
1.5 M Tris-HCL pH 8.8	2.5 mL	2.5 mL	–
0.5 M Tris-HCL pH 6.8	–	–	500 μL
10% SDS	100 μL	100 μL	40 μL
10% APS	100 μL	100 μL	40 μL
TEMED	4 μL	6 μL	4 μL

**CRITICAL:** If you need to prepare all the gels at the same time, you can do so, but do not add 10% SDS, 10% APS and TEMED until you are ready to pour the gels in the casting tray as this will result in gel polymerization and solidification.

**CRITICAL:** If needed to visualize proteins of varied sizes in the same gel, prepare a gradient gel of two different concentrations for example from 8-12%. Prepare 5 mL of each of the gel concentrations in separate conical tubes. Using a 10-mL serological pipette, pipette half the volume from the low gel concentration tube and then add to it the other half from the high gel concentration tube, aspirate air bubble up to the pipette, to allow mixing of the low and high gel concentrations. Slowly pipette the gradient solution into the gel cast, preferably use the serological pipette.

79. Upon gel assembly in the casting tray, pour the prepared resolving gel into the space between the assembled glass plates.

***Note:*** Make sure to leave a space of around 2–4 mL for the stacking gel.

80. Add 1 mL of Isopropanol over the resolving gel to ensure a uniform gel is formed, without any air bubbles.
81. Wait for 30–45 min until gel solidification (can be checked by leaving some gel solution in the tube).
82. Upon solidification of the resolving gel, remove the isopropanol by tilting the apparatus.

***Note:*** Also, you can ensure there is no remaining isopropanol by drying in between the glass plates with filter paper).

83. Pour the stacking gel and insert the 10-well, 1.5 mm comb. Keep the gel to solidify for approximately 30 min, then remove the comb.
84. Electrophoresis and blotting.
  a. Remove the clamp assembly carrying the gel from the gel casting stand and place it into the electrode assembly.
  b. Pour 1× SDS running buffer (into the electrode assembly and in the buffer chamber at a level halfway between the short and the large plates).
    ***Note:*** To prepare 1× SDS running buffer dilute, 10 mL of 10× SDS buffer into 90 mL Milli-Q water, add the water first as SDS form foams.
  c. Initially run the gel for 30 min at low voltages (V) (60 V).
  d. Then apply higher voltages (100–120 V) for the stacking gel.
  e. Once the dye runs off the bottom of the gel, remove the electrode assembly.
  f. To prepare for gel blotting, activate the PVDF membrane as follows:
    i. Soak the membrane in methanol for approximately 2 min.
    ii. Immerse the filter paper in 1× transfer buffer.
    iii. Equilibrate the gel in 1× transfer buffer.
  g. For gel blotting, arrange the gel in the Trans-blot turbo membrane blotter as follows.
    i. Add three layers of filter paper followed by the PVDF membrane.
      ***Note:*** Make notch in one corner to indicate orientation of gel (to indicate which side the ladder is and which way the membrane should face).
    ii. Add the gel followed by other 3 layers of filter paper.
      **CRITICAL:** Ensure that there are no air bubbles while blotting the membranes, by gently using a roller over the membranes.
    iii. Set the transfer conditions as follows: 1.3 A, 25 V for 30 min for the mini gel.
  h. Upon membrane blotting, carefully remove the filter papers and the gel.
  i. Gently, trim the PVDF membrane with blotted proteins.
  j. Place the membrane in a 50-mL conical tubes for blocking in 10 mL of 5% non-fat dry milk in PBS-T (0.5% tween-20 in 1× PBS) for 1 h on a roller at 22°C–25°C.
    **CRITICAL:** Avoid membrane dryness in all steps.
  k. Afterwards, wash the membrane once with 10 mL of 0.5% tween 20 in 1× PBS.
  l. During membrane washing, prepare the primary antibody solution with either the monoclonal VSV M antibody (RRID: AB_2734773) or the monoclonal RV-G primary antibody (RRID: AB_1125351).
    i. Prepare the primary antibody solution at a concentration of 1:2000.
    ii. In a 50 mL falcon tube, mix 2.5 μL of the primary antibody in 5 mL of 5% non-fat dry milk in PBS-T.
  m. Discard the washing solution, then add the primary antibody to the membrane in the falcon tube.
  n. Incubate the membrane with primary antibody at 4°C for 18 ± 2 h on a roller.
    ***Note:*** Ensure to keep rolling the tube while moving it from 22°C-25°C to 4°C to avoid membrane dryness.
  o. Day 6: Wash the membrane 3 times in PBS-T (0.5% tween 20 in 1× PBS), 5 min/wash.
  p. In the meantime, prepare the secondary antibody, polyclonal goat anti-mouse IgG (H&L) HRP at a concentration of 1:3000.
    i. Mix 1.7 μL of the secondary antibody in 5 mL of 5% non-fat dry milk in PBS-T.
  q. Remove the PBS-T, then add the secondary antibody to the membrane, cover the tubes with aluminum foil.
  r. Incubate the membrane in the conical tube for 2 h at 22°C–25°C on a roller.
  s. Remove the secondary antibody and wash the membrane 3 times with PBS-T (0.5% tween 20 in 1× PBS).
85. Immunodetection.
  a. Gently transfer the membrane from the conical tube to a square petri-dish using a forceps.
  b. Add equal volumes of detection reagent 1 and detection reagent 2 to prepare the pierce ECL western blotting substrate.
  c. Cover the Petri dish with aluminum foil and incubate for 1 min.
  d. Place the membrane into the ChemiDoc MP imaging System with a forceps.
    ***Note:*** Adjust the imager to focus on the membrane edges and then start collecting the images using the following protocols: chemi hi sensitivity, chemi hi-resolution and multichannel protocols.
  e. Carry out subsequent image analysis using Image Lab software (Figures 8B and 8C).
    **CRITICAL:** For a loading control, incubate the same protein lysate aliquots with a rabbit polyclonal alpha tubulin antibody then add the goat anti-rabbit IgG H&L, HRP as a secondary antibody.

### Flow cytometry (FC)

**Timing: 4–5 days**

Since both rVSV-dG-RV-G-GFP and rVSV-GFP WT encode GFP which serves as marker of infection, that could be used to determine the virus infection from the GFP expression levels in infected cells. To this end, flow cytometry serves as a useful tool to determine virus infection form the GFP expression levels in infected cells.

86. Day 1: Seed six well plates with BHK-21 cells at a density of 0.3 × 10^6^ cells/well, incubate the plates at 37°C /5% CO_2_ until the following day.
87. Day 2: Upon reaching 70–80% confluency, infect the BHK-21 cells with each of rVSV-dG-RV-G-GFP and rVSV-GFP-WT at MOI of 1.
  a. Prepare triplicate wells of BHK-21 cells infected with either rVSV-dG-RV-G-GFP or rVSV-GFP-WT. Keep 3 uninfected wells to serve as mock control.
  b. Incubate the plates at 37°C for 2 h with shaking every 15–20 min.
  c. After 2 h, remove the virus inoculum, wash the cells with 1× PBS for 3 times, aspirate the PBS and add 1.5 mL of growth medium.
88. Day 4: Thirty hpi, remove the supernatant and wash the cells with 1× PBS.
89. Detach the cells and transfer them into clearly labeled 1.5 mL microcentrifuge tubes for FC analysis.

***Note:*** BHK-21 cells can be easily detached with 1× PBS only, while for other cell lines, trypsin or other reagents may be required such as accutase or versine.

90. Centrifuge the cells at 300 × *g* for 5 min, discard the supernatant and wash the cell pellet once with 1× PBS.
91. After PBS removal, prepare the live dead marker:
  a. Mix 2 μL of LIVE/DEAD Fixable Violet Dead Cell Stain into 2 mL of FCS buffer (2% FBS in 1× PBS).
92. For each specimen, add 200 μL of the prepared live dead marker solution and incubate on ice for 30 min, keep the samples away from light.

**CRITICAL:** If samples will be analyzed on the same day, fixing the cells will not be necessary, however if samples will be analyzed on a different day, fixing the cells in 4% PFA for 1 h, will be required.

**Figure 9:**
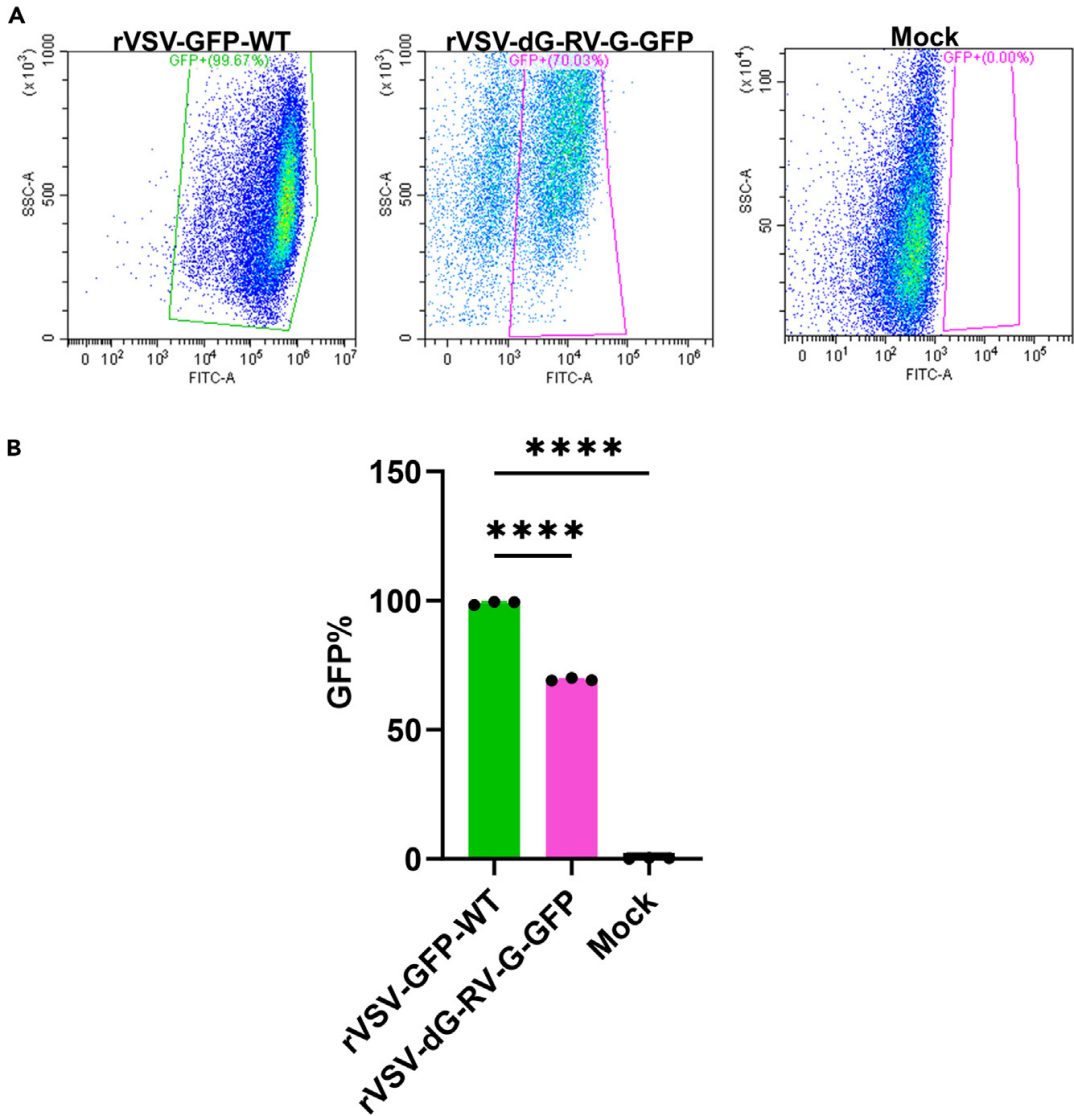
GFP expression on BHK-21 cells infected with rVSV-dG-RV-G-GFP and rVSV-GFP-WT (A) Representative plots for the GFP percentage in infected BHK-21 cells with either rVSV-GFP-WT or rVSV-dG-RV-G-GFP (MOI 1), 30 hpi, along with non-infected cell control. (B) Mean fluorescence intensities of the BHK-21 cell population infected with rVSV-GFP-WT or rVSV-dG-RV-G-GFP at MOI of 1 in comparison to the control cells. These experiments were performed three times independently (*n* = 3). These data represent the average of three biological replicates with S.E.M. indicated. ∗∗∗∗*p* < 0.0001 using one-way ANOVA and student’s t-test.

93. Pellet the cells, then wash once with 1× PBS.
94. Discard the PBS and add 200 μL of 1× permeabilization buffer.
95. Incubate the samples on ice and keep away from light for 15 min.
96. After 15 min, centrifuge the samples and wash the cell pellet once with 1× PBS.
97. Resuspend the pellet in 100 μL of FCS buffer.
98. For FC analysis:
  a. Follow the start-up instruction of the CytoFLEX Flow Cytometer.
  b. Perform QC standardization with flow cytometry beads to calibrate the flow cytometry data.
99. In tube mode, place the tubes corresponding to each of the prepared samples.
  a. Perform from 30,000–50,000 events/sample. Troubleshooting 5.
  b. For each of the samples, plot pseudo color plots as follows (Figure 9A).
    i. First: side scatter (SSC-A) vs. PB450-A for gating live cells only.
    ii. Second: forward scatter area (FSC-A) vs. forward scatter height (FSC-H) for gating singlet cells from live cells.
    iii. Third: side scatter area (SSC-A) vs. FITC-A to gate cell populations expressing GFP, the FITC-A is variable according to the fluorescence tested.
  c. Import the data to USB, then analyze them using CytExpert or FCS Express and plot the data in graph (Figure 9B).

## Expected outcomes

Our protocol describes the procedures to generate rcVSVs. The generated rVSV-dG-RV-G-GFP serves as a reliable model for studying the entry and internalization of rabies and thus allowing to study virus tropism. Since the generated replication competent virus expresses GFP, virus infection can be estimated from the level of GFP expression in the target cells, which can be measured by flow cytometry.

## Limitations

We prepared the rcVSV on BHK-21 cells, so we did not test the rescue process on other cell line. Thus, we recommend preparing the rcVSV on BHK-21 cells. We passaged the generated rcVSV for 3 passages since the main aim was to study virus host interaction. However, if the generated rcVSV would be used for vaccine, it is preferred to do more passages and test the stability of the foreign insert during subsequent passages.

## Troubleshooting

### Problem 1

No colonies observed upon cloning RV-G insert into pVSV-dG-GFP-2.6 (step 14).

### Potential solution

• Try Ligation reactions with different insert: vector ratios such as 1:1 or 5:1. For calculating the molar ratios, use the NEB calculator.
• Incubate the LB plates at 30°C for 24–48 h.

### Problem 2

No GFP observed upon co-transfecting pVSV-dG-RV-G-GFP and helper plasmids (step 31).

### Potential solution

• Try different plasmid ratios.
• For splitting the BHK-21 cells the day before co-transfection, use 1× PBS instead of trypsin.

### Problem 3

Cell death upon inoculating the rescued rVSV-dG-RV-G-GFP (step 32).

### Potential solution

• For further passaging of rVSV-dG-RV-G-GFP, try different concentrations during the inoculation of the rescue virus rVSV-dG-RV-G-GFP, since high concentrations might cause cell death, while low concentration might be insufficient.

### Problem 4

Absence of well-defined plaques (step 54).

### Potential solution

• Test 2 different overlay media, semi-solid medium (such as 3% CMC) and solid medium (such as 1.6% agarose) and compare them in terms of defined plaque formation.

### Problem 5

No difference observed in the GFP % observed between infected and uninfected cells with rVSV-dG-RV-GFP (step 99).

### Potential solution

• It is important to maintain a similar number of events for all samples to allow uniform comparative data analysis.
• It is crucial to include a non-fluorescent control (mock cells), to analyze the fluorescent samples accordingly.

## Resource availability

### Lead contact

Further information and requests for resources and reagents should be directed to and will be fulfilled by the lead contact, Prof Muhammad Munir (muhammad.munir@lancaster.ac.uk).

### Technical contact

Questions about the technical specifics of performing the protocol should be directed to and will be answered by the technical contact, Dr Manar E. Khalifa (manaressam13@gmail.com).

### Materials availability

The pVSV-dG-RV-G-GFP plasmid and the rVSV-dG-RV-G-GFP generated in this study are available upon request.

### Data and code availability

This study did not generate any unique datasets or code.

## Acknowledgments

This study was funded by the 10.13039/501100000268Biotechnology and Biological Sciences Research Council (BBSRC) (BB/M008681/1 and BBS/E/I/00001852) and the British Council (172710323 and 332228521). The PhD studies of M.E.K. have been financially supported by Newton-Mosharafa Fund (Bureau ID: NMM11/19) and the Egyptian Ministry of Higher Education and Scientific Research, Cultural Affairs and Mission Sector, Egypt. The authors would like to thank Kerafast for providing original working plasmids and Luis Martinez-Sobrido from Texas Biomedical Research Institute, USA, for providing https://www.txbiomed.org/scientists/luis-martinez-sobrido-ph-d/pCITE-GFP and pCAGG-GFP plasmids. Graphical abstract/figures were created using BioRender.com.

## Author contributions

M.E.K. and M.M. wrote the protocol. M.E.K. prepared the figures. M.E.K. and M.M. reviewed and approved the final manuscript.

## Declaration of interests

The authors declare no competing interests.
