# Supplementary material for: Protocol for constructing and characterizing recombinant vectored vaccines for rabies virus
Source: STAR Protoc. 2024 Oct 17;5(4):103392. doi: 10.1016/j.xpro.2024.103392 (PMC11513535; doi:10.1016/j.xpro.2024.103392)
Supplement: Document S1. Figure S1 [file mmc1.pdf]

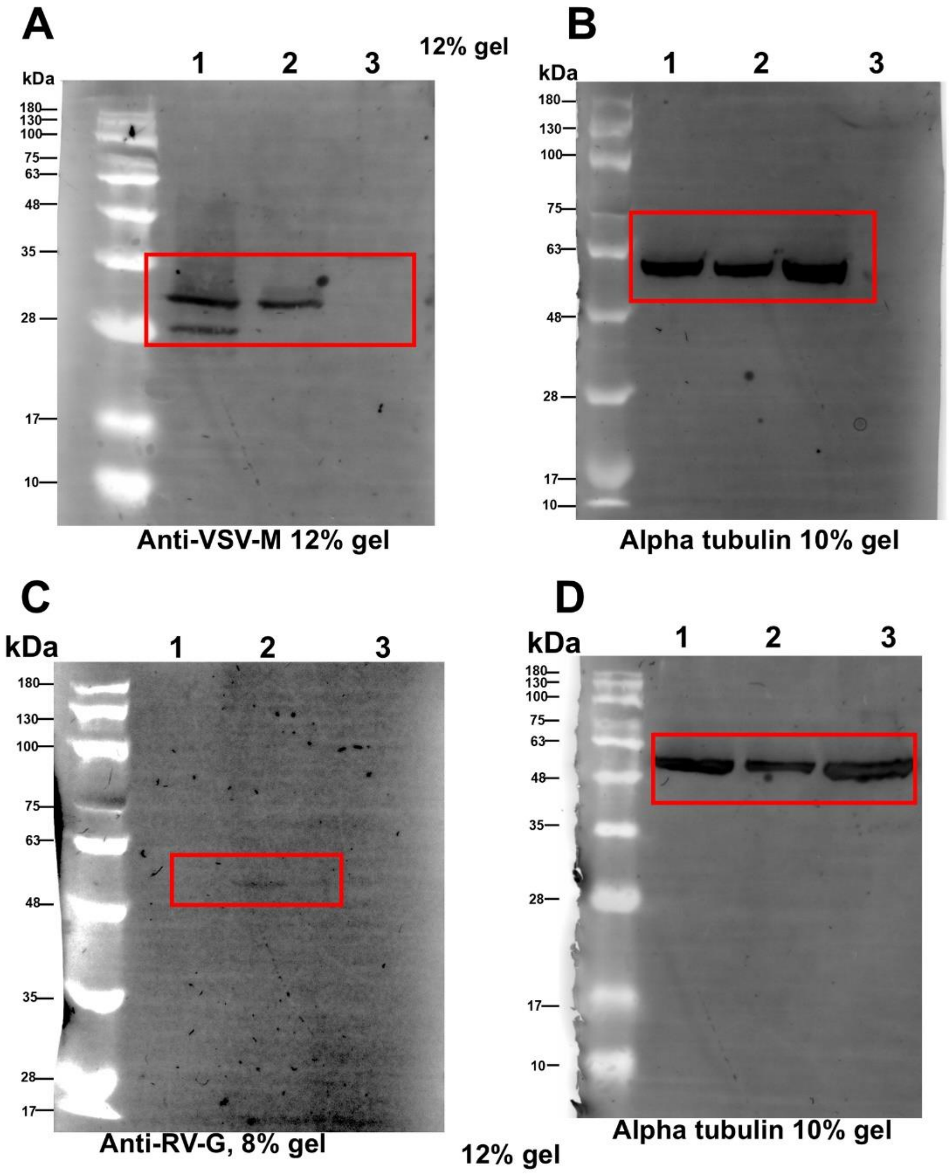

Supplementary Figure 1. Uncropped images of blots used in Figure 8. (A) Blot stained against the VSV-M antibody. (B) Blot stained against the alpha tubulin antibody (C). Blot stained against the RV-G antibody. (D) Blot stained against the alpha tubulin antibody. Red boxes refer to the region used for figures. Position of the molecular mass markers is shown in kDa. 1; rVSV-dG-GFP-WT, 2; rVSV-dG-RV-G-GFP, 3; Cell lysate.
